# Human endometrium-derived mesenchymal stem/stromal cells application in endometrial-factor induced infertility

**DOI:** 10.3389/fcell.2023.1227487

**Published:** 2023-09-05

**Authors:** Raminta Bausyte, Brigita Vaigauskaite - Mazeikiene, Veronika Borutinskaite, Elvina Valatkaite, Justinas Besusparis, Ruta Barbora Valkiuniene, Edita Kazenaite, Diana Ramasauskaite, Ruta Navakauskiene

**Affiliations:** ^1^ Life Sciences Center, Department of Molecular Cell Biology, Institute of Biochemistry, Vilnius University, Vilnius, Lithuania; ^2^ Center of Obstetrics and Gynaecology of Institute of Clinical Medicine, Faculty of Medicine, Vilnius University, Vilnius, Lithuania; ^3^ Faculty of Medicine, Vilnius University, Vilnius, Lithuania; ^4^ National Center of Pathology, Vilnius University Hospital Santaros Klinikos, Vilnius, Lithuania; ^5^ Faculty of Medicine, Vilnius University Hospital Santaros Klinikos, Vilnius University, Vilnius, Lithuania

**Keywords:** infertility, endometrium-derived mesenchymal stem cells, stromal cells, endometrial-factor, mechanical injury, chemotherapy, histology, genes

## Abstract

Endometrial-factor induced infertility remains one of the most significant pathology among all fertility disorders. Stem cell-based therapy is considered to be the next-generation approach. However, there are still issues about successfully retrieving human endometrium-derived mesenchymal stem/stromal cells (hEnMSCs). Moreover, we need to establish a better understanding of the effect of hEnMSCs on the endometrial recovery and the clinical outcome. According to these challenges we created a multi-step study. Endometrium samples were collected from females undergoing assisted reproductive technology (ART) procedure due to couple infertility. These samples were obtained using an endometrium scratching. The hEnMSCs were isolated from endometrium samples and characterized with flow cytometry analysis. Groups of endometrium injured female mice were established by the mechanical injury to uterine horns and the intraperitoneal chemotherapy. The hEnMSCs suspension was injected to some of the studied female mice at approved time intervals. Histological changes of mice uterine horns were evaluated after Masson’s trichrome original staining, hematoxylin and eosin (H&E) staining. The fertility assessment of mice was performed by counting formed embryo implantation sites (ISs). The expression of fibrosis related genes (*Col1a1, Col3a1*, *Acta2*, and *CD44*) was evaluated by the reverse transcription—quantitative polymerase chain reaction (RT-qPCR). Results showed that endometrium scratching is an effective procedure for mesenchymal stem/stromal cells (MSCs) collection from human endometrium. Isolated hEnMSCs met the criteria for defining MSCs. Moreover, hEnMSCs-based therapy had a demonstrably positive effect on the repair of damaged uterine horns, including a reduction of fibrosis, intensity of inflammatory cells such as lymphocytes and polymorphonuclear cells (PMNs) and the number of apoptotic bodies. The injured mice which recieved hEnMSCs had higher fertility in comparison to the untreated mice. Gene expression was reflected in histology changes and outcomes of conception. In conclusion, hEnMSCs demonstrated a positive impact on endometrium restoration and outcomes of endometrial-factor induced infertility. Further exploration is required in order to continue exploring the multifactorial associations between stem cell therapy, gene expression, endometrial changes and reproductive health, so we can identify individually effective and safe treatment strategies for endometrial-factor induced infertility, which is caused by mechanical effect or chemotherapy, in daily clinical practise.

## Introduction

Infertility has become a significant health problem. Overall, approximately one in six heterosexual couples (or 8%–12% women aged 20–44 years) around the world may experience difficulty getting pregnant (ESHRE Capri Workshop Group, 2001). Studies on the mechanisms of fertility disorders have identified human endometrial tissue as one of the key elements in reproductive health field.

Regardless of the method of conception, endometrial receptivity—the ability of endometrium to successfully attach and maintain the embryo—is an essential condition for conception and further pregnancy development (Heger et al., 2012; Lessey and Young, 2019). Unfortunately, the endometrial receptivity can be impaired by the endometrial dysfunction such as intrauterine adhesions, Asherman’s syndrome, a persistently thin endometrium or an endometrial atrophy. In this way, these pathologies have been associated with infertility (Liu et al., 2018; Mahutte et al., 2022), increased risk of miscarriage (Yuan et al., 2016; Bu et al., 2020), ectopic pregnancy (Rombauts et al., 2015; Liu et al., 2020), placenta previa (Jing et al., 2019), low birthweight (Moffat et al., 2017; Oron et al., 2018; Ribeiro et al., 2018; Guo et al., 2020) and other obstetric complications (Oron et al., 2018).

The main treatment strategy for the mentioned endometrial dysfunction consists of medical therapy (estrogen therapy, vitamin E, pentoxifylline, L-arginine, aspirin, tamoxifen, sildenafil), surgical procedures (dilatation and curettage, hysteroscopy), devices and materials of re-adhesion prevention (intrauterine device, uterine stent, Foley’s catheter, adhesion barriers), autologous cellular therapy (intrauterine infusion of granulocyte colony stimulating factor or autologous platelet-rich plasma) and assisted reproductive technology (ART) procedures (AAGL Elevating Gynecologic Surgery, 2017; Ranisavljevic et al., 2019; Santamaria et al., 2020; Mouanness et al., 2021; Strug and Aghajanova, 2021). Although multiple approaches to treatment are available, the overall curative outcome is quite inconsistent. Therefore, stem cell-based therapy is being explored as a potential next-generation approach for endometrial-factor induced infertility.

Stem cells have been recognized as a potential treatment strategy for endometrium pathology caused infertility due to self-renewal and multilineage differentiation, disruption of fibrosis and promotion of angiogenesis, immune regulation and paracrine stimulation (Fossett et al., 2012; Song et al., 2021; Wu et al., 2022). Much attention has been paid to mesenchymal stem/stromal cells (MSCs) as a potential therapy tool due to their properties in contrast to other stem cell types: MSCs are a well-characterized population of adult stem cells, with no ethical or moral issues attached to their usage; they have a wide range of differentiation potential; they can be easily grown and multiplied in culture to create the required amount, without losing differentiation potential; they have a low immunogenicity; they lack tumorigenesis properties; they can act through different mechanisms and, they are even easily available commercially (Tipnis et al., 2010; Lee, 2018; Lukomska et al., 2019; Musiał-Wysocka et al., 2019; Wang et al., 2019). MSCs can be isolated from different adult tissues (bone marrow, adipose tissue, dental pulps, peripheral blood, skin, synovial fluid, muscle, menstrual blood or endometrial tissue) (Taylor, 2004; Mints et al., 2008; Figueira et al., 2011; Phermthai et al., 2016; Chen et al., 2019; Fuoco et al., 2020) and neonatal tissues (umbilical cord, Wharton’s jelly, amniotic fluid, placenta or amniotic membrane) (Phermthai et al., 2010; Figueira et al., 2011; Zhang et al., 2018; Ouyang et al., 2020; Lin et al., 2022). While the best-known and most prevalent sources of MSCs are bone marrow, adipose tissue and umbilical cord, increasing evidence from basic and preclinical studies suggests that human endometrium-derived mesenchymal stem/stromal cells (hEnMSCs) show great promise as an autologous source for regenerative medicine, including reproductive health (Abuwala and Tal, 2021). While quite a lot of knowledge has been accumulated about hEnMSCs, their role in the repair process of the endometrium and the impact on fertility disorders treatment, remains elusive.

The aim of the present study is to evaluate the endometrium as a potential source of multipotent MSCs, while specifying hEnMSCs collecting methods, and investigating their impact on endometrial-factor induced infertility according to the multifactorial background along with an analysis of histological findings, gene expression and fertility assay.

## Materials and methods

### Patient recruitment

The study “Therapeutic potential of human endometrial stem cells” was approved by the Ethics Committee of Biomedical Research of Vilnius Region, No. 158200-18/7-1049-550 (29/06/2018). All endometrial tissue samples were collected during routine procedure at Vilnius University Hospital Santaros Klinikos Obstetrics and Gynaecology Center Santaros Fertility Center. The female subjects of the present study signed consent forms to confirm their agreement to providing their endometrium specimens.

Endometrial tissue samples were taken from a total 20 females undergoing ART procedures due to couple infertility. The inclusion criteria were as follows: (a) age of female at the time of enrollment is 18–45 years; (b) the minimum duration of infertility—1 year; (c) female is undergoing ART procedures due to couple infertility; (d) female confirms the participation in the study and signed informed consent. The exclusion criteria were as follows: (a) confirmed oncological disease for female during the last 3 years; (b) female smokes or is addicted to alcohol or other substances; (c) pregnancy is contraindicated for female; (d) female is diagnosed with uncontrolled endocrine or other medical conditions, such as hyperprolactinemia or thyroid diseases; (e) female was diagnosed with mental disorders or illness in the past medical history.

A reproductive medicine professional collected the specimens using 3 mm wide pipelle, endometrial sampling catheter, while performing a scratching procedure on the inner wall of uterine lining. The procedure was carried out once on Day 20–22 of the menstrual cycle prior to *in vitro fertilization* (IVF)/intracytoplasmic sperm injection (ICSI) procedure.

### Isolation and cultivation of human endometrium-derived mesenchymal stem/stromal cells

The isolation of hEnMSCs was performed according to Tavakol et al. (2018). Shortly, endometrial tissue samples were washed with Hank’s media, cut into small pieces and incubated with collagenase I (1 mg/ml) for approximately 60 min. The growth medium was then added and the sample was passed through a 70 µm Falcon cell strainer. Passed cells were centrifuged and purified from red blood cells (RBCs) by using Ficoll gradient. Isolated cells were cultured in a growth medium DMEM/F12 (DMEM, Dulbecco’s Modified Eagle Medium/Nutrient Mixture F-12) supplemented with 10% Fetal Bovine Serum (FBS), 100 U/mL penicillin and 100 μg/mL streptomycin (Gibco, Thermo Fisher Scientific, Waltham, MA, United States) at 37°C in a 5% CO_2_ atmosphere with humidity. Cells were plated into T75 cm^2^ culture flasks at a density of 0.5–0.6 × 10^6^ cells. The medium was replaced every 2–3 days and cells were replated according to their confluence. Cell viability was evaluated during passaging using Neubauer’s chamber and Trypan Blue test. Preparation of hEnMSCs for the injection into female mice was done by washing cells twice with sterile phosphate-buffered saline (PBS) solution.

### Flow cytometry analysis

To determine hEnMSCs surface markers, cells were collected (about 0.1 × 10^6^ cells for one marker), centrifuged at 500 *g* speed for 5 min and washed twice with 1xPBS and 1% Bovine serum albumin (BSA) solution. Washed cells were then incubated for 30 min in the dark on ice with appropriate antibodies against cell surface markers (CD44, CD146, CD166, CD140b, CD34, CD45, HLA-DR). After that, the labelled cells were washed twice with PBS +1% BSA solution by centrifuging at 550 g for 5 min, 4°C. Since flow cytometry analysis was not done immediately, cells were fixated with 2% paraformaldehyde solution and later analyzed with Partec flow cytometer (Sysmex Corporation, Cobe, Hioko, Japan). Results were processed with Flowing Software 2 software.

### Animal study

Animal testing “Possibilities of stem cells application for restoration of endometrium” was approved by the State Food and Veterinary Service of the Republic of Lithuania. Veterinary approval for that study was No. G2-115 (20/05/2019).

Procedures with animal were performed by trained specialists using appropriate equipment.

A total of 58 NOD. CB-17-Prkdc scid/Rj female mice, 6 weeks of age, 18 g ± 0.5 g were purchased from JANVIER LABS (France) and allowed to adapt to the new environment for at least a week. The mice were maintained in specific-pathogen free conditions.

The anesthetized mice were euthanized by cervical dislocation procedure. Mice were removed from their cages, anesthetized by 1%–3% isoflurane inhalation (100 mg/g, Isoflurin, Vetpharma, Spain) and gently restrained while resting on the operating tables with aseptic pads. Cervical dislocation was performed mechanically and resulted in euthanasia within approximately 10 s.

The Control mice group *(n = 11)* did not receive any interventions. The group of female mice with mechanically damaged endometrium and the chemotherapy-induced female mice were designed by the following procedures.

#### A design of mechanically damaged endometrium model in female mice

To construct a mechanically damaged endometrium model, 23 female mice were anesthetized by 1%–3% isoflurane inhalation (100 mg/g, Isoflurin, Vetpharma, Spain). Once the mice had no limb reaction, they were placed in a supine position with the limbs gently fixed on the operating table with aseptic pads. The operation area was disinfected by 75% alcohol, and a longitudinal incision of 1.5 cm was made in the low midline abdomen. Ophthalmic forceps were used to lift the skin, and ophthalmic scissors were used to cut the skin layer-by-layer longitudinally. A 21-gauge needle was inserted into the connection between the left and right uterine horns, and the right horn was scratched back and forth carefully until the uterus became hyperemic (approximately 10 times) while continuing to maintain the wholeness of the horn. The abdominal cavity was subsequently closed. After 7 days, the affected female mice were divided into three groups: a) the mice group which was euthanized right away and its uterine horns were sent for further analysis (the MechI mice group, *n = 8*); b) the mice group which was housed together with male mice for 2 weeks *(n = 5)*; and c) the mice group which was being prepared for the investigation of hEnMSCs treatment impact on the injured endometrium (the MechI-hEnMSCs mice group, *n = 10*). A few additional steps were required to achieve this goal. First, the abdominal cavity of the mice in the MechI-hEnMSCs group was reopened according to the previous protocol. When in the back of the bladder, the bilateral uterus was touched, then straight forceps were used to clamp the right horn in the upper third and in the lower third. hEnMSCs (0.5 × 10^6^ cells/20 μl PBS) suspension was injected directly to the right horn. Subsequently, the abdominal cavity was closed. After 7 days, the MechI-hEnMSCs group of mice were divided into another two groups: a) mice that were euthanized right away with their uterine horn sent for further analysis *(n = 5)*; and b) mice that were housed together with male mice for 2 weeks *(n = 5)*. At the end of the study, the mated female mice were euthanized to evalute their fertility by counting embryo implantation sites (ISs). The number of embryo ISs in uterine horns was visually recorded after uterine horns dissection.

#### A design of chemotherapy-induced female mice model

To construct a chemotherapy-induced female mice model, 24 mice were injected once intraperitoneally with cyclophosphamide 120 mg/kg and busulfan 30 mg/kg. After 7 days, the affected female mice were divided into three groups: a) the mice group which was euthanized right away with its uterine horn were sent for further analysis (the CheI mice group, *n = 9*); b) the mice group housed together with male mice for 2 weeks *(n = 5)*; and c) the mice group prepared for the investigation of hEnMSCs treatment impact on injured endometrium (the CheI-hEnMSCs mice group, *n = 10*). The anesthesia and the operation procedure for female mice with the aim to infused hEnMSCs suspension was performed according to procedures described in the section “A design of mechanically damaged endometrium model in female mice”. After 7 days, the CheI-hEnMSCs mice were divided into two groups: a) mice that were euthanized right away with their uterine horn sent for further analysis *(n = 5)*; and b) mice that were housed together with male mice for 2 weeks *(n = 5)*.

A fertility assessment of the female mice was performed after 14 days. The studied mice were then euthanized to evalute their fertility by counting embryo ISs. The number of embryo ISs in uterine horns was visually recorded after uterine horns dissection.

### RNA isolation from female mice uterine horns and gene expression analysis using RT-qPCR

*Quick*-DNA/RNA Miniprep Kit (Zymo Research, CA, United States) was used to facilitate the total RNA extraction from mice uterine horns. Frozen tissue was first submerged in liquid nitrogen and ground to a powder using a mortar and pestle. Then powder was mixed with about 600 µl of DNA/RNA Lysis Buffer, pipetted and transferred to a Zymo-Spin™ Column and centrifuged at 13,000 g for 1 min at room temperature. The following isolation steps were done in accordance with manufacturer’s recommendations to ensure high yield and purity of the RNA samples. cDNA was synthesized using Luna^®^ Universal One-Step the reverse transcription—quantitative polymerase chain reaction (RT-qPCR) Kit (New England Biolabs, Ipswich, MA, United States) in accordance with the manufacturer’s recommendations. cDNA was then amplified by RT-qPCR using Luna^®^ Universal qPCR Master Mix (New England Biolabs, Ipswich, MA, United States) in accordance with the manufacturer’s guidelines. RT-qPCR was performed using Rotor-Gene 6,000 Real-time Analyzer (Corbett Life Science, QIAGEN, Hilden, Germany) and cycling conditions were set as follows: initial denaturation 95°C 1 min (1 cycle), denaturation 95°C 15 s, primer Tm 60°C 30 s (40 cycles). The sequences of forward and reverse primers used in this study are detailed in Table 1. Relative gene expression of *Col1a1 (Collagen, type I, alpha 1), Col3a1 (Collagen, type III, alpha 1), Acta2 (Actin alpha 2, smooth muscle)* and *CD44* was calculated using ΔΔCt method and mRNA levels were normalized according to *18S* expression.

**TABLE 1:** List of primers used for RT-qPCR analysis.

Gene name	Forward primer (5‘-3‘)	Reverse primer (5‘-3‘)
*18S*	GGA​AGG​GCA​CCA​CCA​GGA​GT	TGC​AGC​CCC​GGA​CAT​CTA​AG
*Col1a1*	TCA​CCA​AAC​TCA​GAA​GAT​GTA​GGA	GAC​CAG​GAG​GAC​CAG​GAA​G
*Col3a1*	ACA​GCA​GTC​CAA​CGT​AGA​TGA​AT	TCA​CAG​ATT​ATG​TCA​TCG​CA
*Acta2*	TTG​CTG​ACA​GGA​TGC​AGA​AGG​AGA	ATC​TGC​TGG​AAG​GTA​GAC​AGC​GAA
*CD44*	ATC​AGC​AGA​TCG​ATT​TGA​ATG​TAA	CAT​TTC​CTT​CTA​TGA​ACC​CAT​ACC

### Evaluation of uterine horns histology

Samples of uterine horns collected from female mice were fixed in 10% neutral buffered formalin with subsequent paraffin embedding. 3 μm-thick sections were stained with hematoxylin and eosin (H&E) and Masson’s trichrome original in accordance with standard protocol. Digital whole slide images were recorded using a ScanScope XT Slide Scanner (Leica Aperio Technologies, Vista, CA, United States) under ×20 objective magnification (0.5-μm resolution) and subsequently subjected to digital image analysis by using HALOTM software (version 3.0311.174; Indica Labs, Corrales, New Mexico, United States). The positive pixel counts based HALO Area Quantification v2.1.3 algorithm was calibrated to recognize fibrosis areas and other surrounding tissues. Pixel intensity values were manually determined by the pathologist. Examples of image analysis output are presented in Figure 4 and Figure 6. The repeated manual measurements of the thickness of the total uterine horn wall and its separate layers (10 measurements per each tissue sample) were performed by using HALOTM software.

### Statistical analysis

Statistical analysis was performed using GraphPad Prism version 8.0.1 for Windows, GraphPad Software, San Diego, California United States, www.graphpad.com. Data in graphs are represented as mean ± standard deviation (S.D.), while triangular data points indicate outliers determined by ROUT (Q = 5%). The statistical significance of difference was calculated using Mann-Whitney U test, Kruskal-Wallis test and Bonferroni test, significance was set at *p* ≤ 0.05 (*), *p* ≤ 0.01 (**), *p* ≤ 0.001 (***).

## Results

**FIGURE 1:**
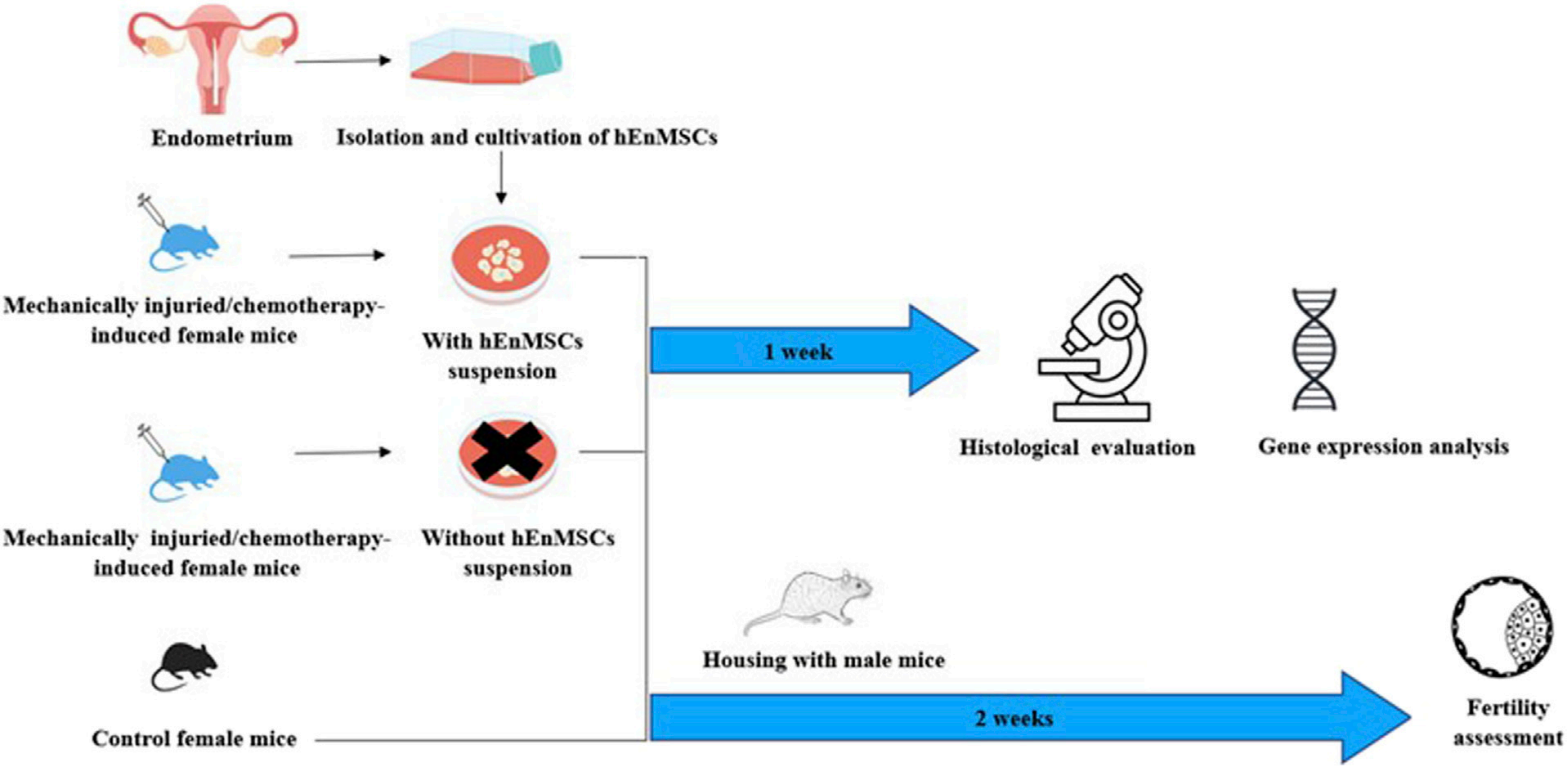
Schematic view of the study performed on different female mice groups: the Control mice group, the mechanically injuried endometrium mice group and the chemotherapy-induced mice group *(Adopted images from Shutterstock system)*.

In this work, the effect of cells isolated from human endometrium on the female mice of mechanically damaged and chemotherapy-induced model was investigated (Figure 1). During the study, cells isolated from endometrium were characterized using flow cytometry. After, the cells were subsequently injected into the uterine horns of injured female mice. Changes in the uterine horn tissue were investigated by histological analysis, expression of genes related to the process of fibrosis. The therapeutic effect of cells to restore infertility has also been established.

### Characterization of hEnMSCs

**FIGURE 2:**
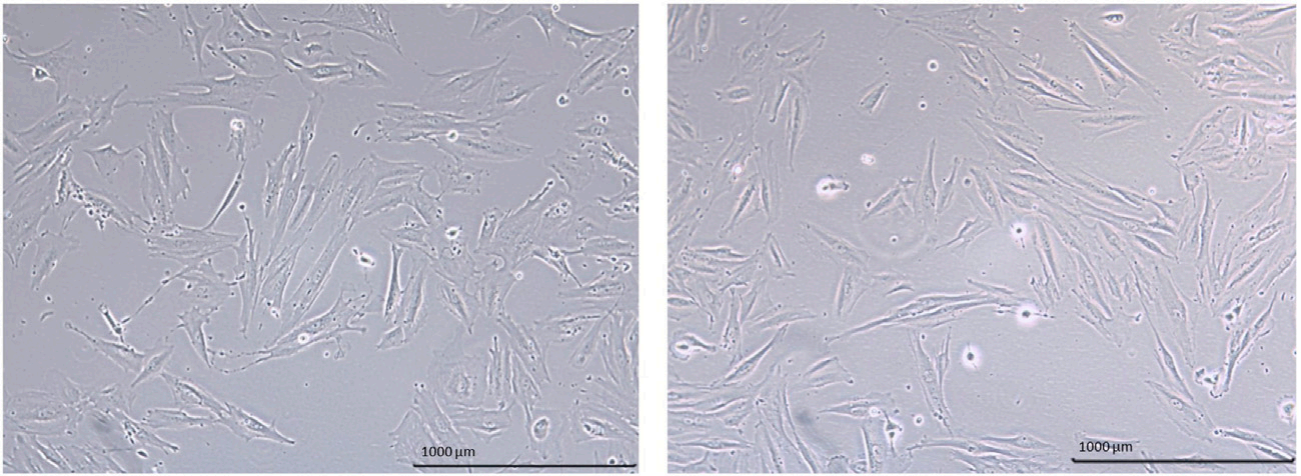
Morphology of hEnMSCs early passages. Images were taken with ×10 objective, scale bar—1000 μm.

The morphology of hEnMSCs was observed during cultivation and prior to the injection into different female mice models (Figure 2). The images indicate that cells had spindle-shaped morphology, were adherent and formed a consistent monolayer. The cells were cultured and further characterized at early passages.

**FIGURE 3:**
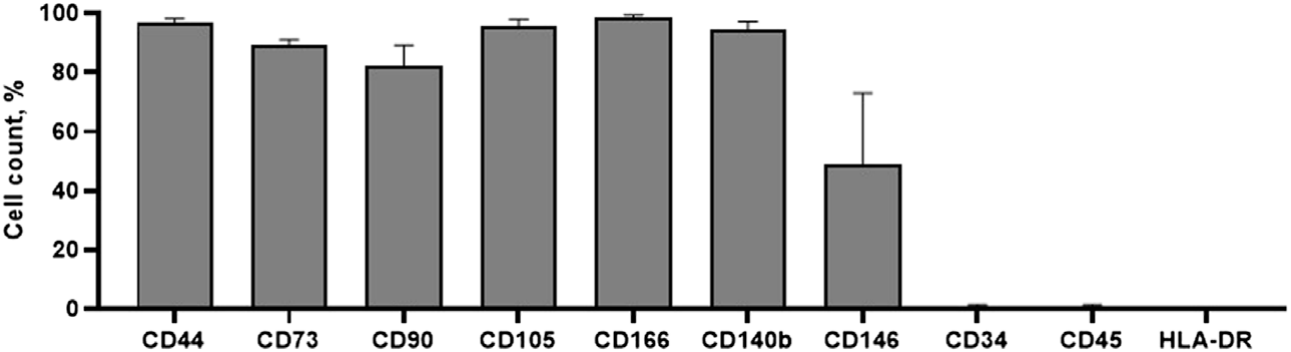
Characterization of hEnMSCs by the expression of surface markers. The cells were tested against mesenchymal stem cells markers: CD44, CD73, CD90, CD105, CD166; endometrial stromal cells markers: CD140b, CD146; hematopoietic and immune cells markers: CD34, CD45, HLA-DR. Analysis was performed using flow cytometry. Results are presented as mean ± S.D.

Cell surface analysis by flow cytometry was carried out to ensure that our isolated cells corresponded with basic MSCs properties. We have founded that hEnMSCs were identified as positive with CD44, CD73, CD90, CD105, CD146, CD166 and CD140b cell surface markers (Figure 3). CD44 and CD166 were determined to be highly positive, exceeding 95%. Positive expression of CD146 and CD140b markers are characteristic to endometrial stromal cells and positive expression of CD44, CD73, CD90, CD105 surface markers confirms mesenchymal origin of stem cells. Moreover, hEnMSCs did not express hematopoietic cell surface markers CD34, CD45 and HLA-DR, which were all measured at less than 1%. Histograms of analyzed surface markers are presented in Supplementary Figure S1. In order to further characterize hEnMSCs by their differentiational abilities, we induced hEnMSCs to specialize into adipogenic, osteogenic, myogenic, neurogenic, and chondrogenic lineages in our previous research (Žukauskaitė et al., 2023). According to our results, we showed that hEnMSCs were able to successfully differentiate into adipogenic, osteogenic, neurogenic, and chondrogenic directions.

### Establishment a multi-step analysis of different female mice models

The mechanically damaged endometrium model of NOD. CB-17-Prkdc scid/Rj female mice was established via endometrial scratching of the right uterine horn of female mice. Meanwhile, chemotherapy-induced female mice model was established using intraperitoneally chemotherapy. The Control mice model did not receive any interventions.

Uterine horns were collected and histologically evaluated 7 days after the endometrium injury (the MechI mice group) or intraperitoneally chemotherapy induction (the CheI mice group), and 7 days after hEnMSCs-treatment usage for endometrium regeneration in the MechI-hEnMSCs mice group and the CheI-hEnMSCs mice group, accordingly. Masson’s trichrome original staining and H&E staining was used to evaluate histological changes in layers of uterine horns: the total wall, the myometrium-endometrium, the myometrium and the endometrium.

A fertility assessment was performed by visually counting of embryo ISs after 14 days of housing female mice and male mace together in these groups: female mice with mechanically damaged endometrium (MechI) or chemotherapy induction (CheI) and female mice with mechanically damaged endometrium or chemotherapy induction which received hEnMSCs-treatment usage for endometrium regeneration—the MechI-hEnMSCs mice group and the CheI-hEnMSCs mice group, accordingly. In the mechanically injured endometrium model, we counted the number of embryo ISs only in the one uterine horn which was affected. Meanwhile, in the chemotherapy-induced female mice, the number of embryo ISs was counted in two uterine horns due to the systematic effect of the medication.

Expression analysis of genes involved in fibrosis process *(Col1a1, Col3a1, Acta2 and CD44*) was performed with study’s female mice groups using RNA extracted from their uterine horn tissue samples.

### Fibrotic transformation under the influence of hEnMSCs-treatment in mechanically damaged endometrium model of female mice

**FIGURE 4:**
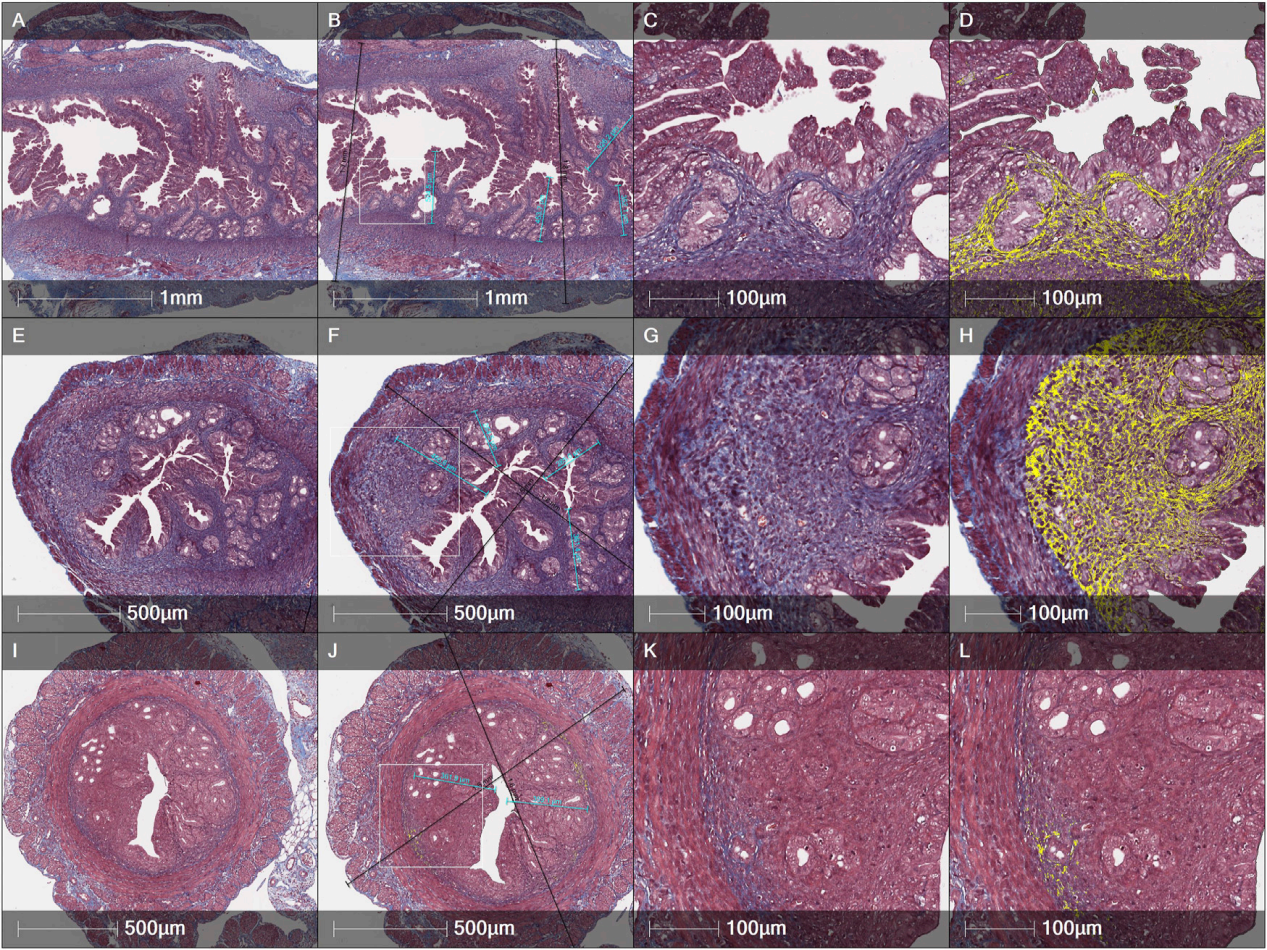
Histological evaluation of the thickness of endometrial and the total wall of uterine horn and fibrosis spread by Masson’s trichrome original staining in different female mice groups: **(A–D)**—the Control, **(E–H)**—the MechI, (**I–L)**—the MechI-hEnMSCs. Figures **(B, F and J)** indicating measurements of endometrial thickness *(light blue)* and the total wall of uterine horn *(black)*. Fibrosis mark-up area *(in yellow)* on digitized slide obtained by automated image analysis Area Quantification algorithm **(D, H, L)**. Scale bar—1 mm, 500 μm and 100 μm. Figures **(B, F and J)**: white rectangle indicates higher magnification reference frames for Figures (**C, D, G, H, K, L)**.

**FIGURE 5:**
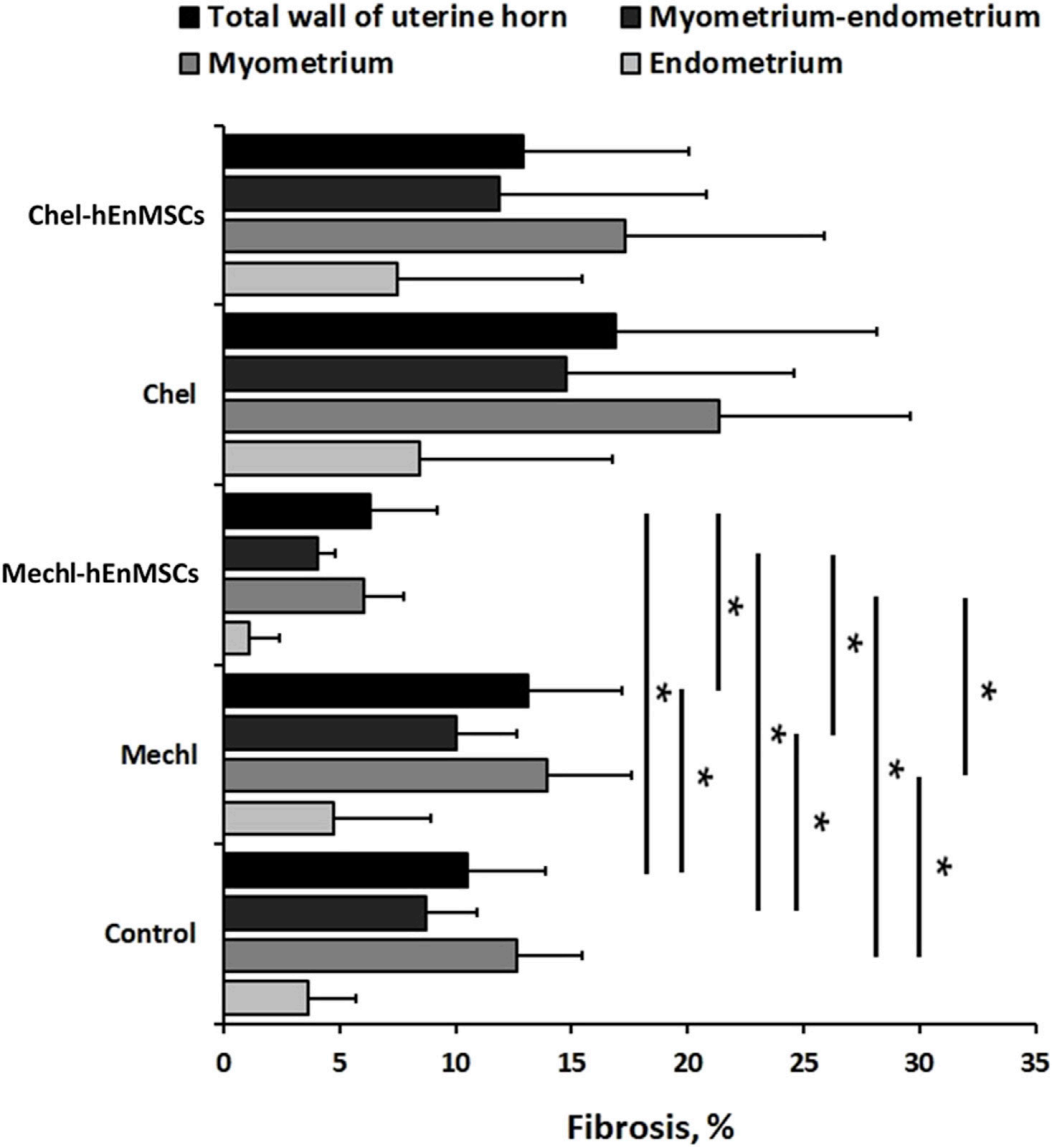
Statistical analysis of fibrotic transformation in different layers of uterine horns (the total wall of uterine horn, the myometrium-endometrium, the myometrium, and the endometrium). Uterine horns samples were collected from different female mice groups: the Control *(n = 4)*, the MechI *(n = 6)*, the MechI-hEnMSCs *(n = 4)*, the CheI *(n = 3)* and the CheI-hEnMSCs *(n = 3)*. Results are presented as mean and ±S.D. Statistical significance was evaluated using the Kruskal-Wallis test and Bonferroni test, where *denotes *p* ≤ 0.05.

An evaluation of fibrosis in different layers of uterine horns (the total wall, the myometrium-endometrium, the myometrium and the endometrium) was performed in 3 different female mice groups (the Control, the MechI and the MechI-hEnMSCs) using Masson’s trichrome original staining (Figures 4D, H, L) and was subsequently statistically analyzed (Figure 5).

Statistical analysis showed significant differences in the fibrotic area between the Control mice group (Figure 4D), the MechI mice group (Figure 4H) and the MechI-hEnMSCs mice group (Figure 4L) in separate layers of mechanically injured uterine horns such as the total wall of uterine horn (Kruskal-Wallis; H = 8.38; df = 2; *p* = 0.02), the myometrium-endometrium (Kruskal-Wallis; H = 7.28; df = 2; *p* = 0.03) and the myometrium (Kruskal-Wallis; H = 7.53; df = 2; *p* = 0.02) (Figure 5). Conversely, the endometrial fibrosis was significant similar (Kruskal-Wallis; H = 5.58; df = 2; *p* = 0.06), although this related to the tendencies of the widest fibrotic area in the MechI mice group (Figure 4H) and the narrowest fibrotic area in the MechI-hEnMSCs mice group (Figure 4L) (Figure 5). Overall, the lowest spread of fibrosis was observed of all layers of uterine horns in MechI-hEnMSCs mice group (Figure 4L)—the total wall 6.3% (±1,3%), the myometrium-endometrium 4.1% (±2.9%), the myometrium 6.0% (±0.8%) and the endometrium 1.1% (±1.7%) compared to other study’s groups (Figure 5).

The Bonferroni *post hoc* test revealed that the percentage of fibrosis spread in the total wall and its separate layers—the myometrium and the myometrium-endometrium—was significantly higher in Mech I mice group (Figure 4H) compared to MechI-hEnMSCs mice group (Figure 4L) (Bonferroni test in all cases; *p* < 0.05), although the differences in mean values between the Control mice group (Figure 4D), the MechI mice group (Figure 4H) and the MechI-hEnMSCs mice group (Figure 4L) were not statistically significant (in all cases Bonferroni test; *p* > 0.05) (Figure 5).

### Fibrotic transformation under the influence of hEnMSCs-treatment in chemotherapy-induced female mice model

**FIGURE 6:**
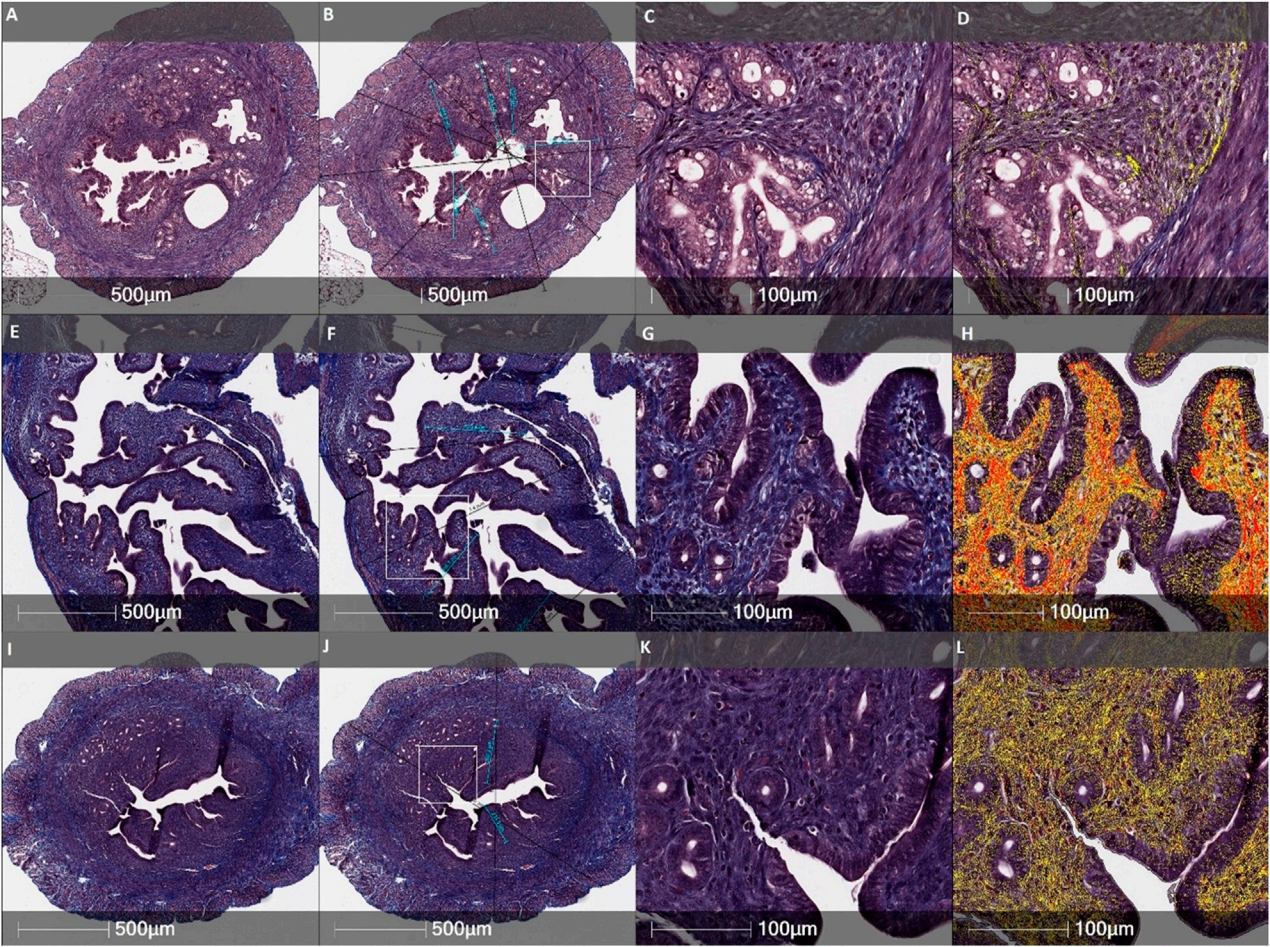
Histological evaluation of the thickness of endometrial and the total wall of uterine horn and fibrosis by Masson’s trichrome original staining in different female mice groups: **(A–D)**—the Control mice group, **(E–H)**—the CheI mice group, **(I–L)**—the CheI-hEnMSCs mice group. Figures **(B, F and J)** indicate measurements of the endometrial thickness *(light blue)* and the total diameter of uterine horn *(black)*. Fibrosis mark-up area *(in yellow and red)* on digitized slide obtained by automated image analysis Area Quantification algorithm **(D, H, L)**. Scale bar −500 μm and 100 μm. Figures **(B, F and J)**: white rectangle indicates higher magnification reference frames for Figures (**C, D, G, H, K, L)**.

Evaluation of fibrosis in different layers of uterine horns (the total wall of uterine horn, the myometrium-endometrium, the myometrium and the endometrium) was performed in 3 different female mice groups (the Control, the CheI and the CheI-hEnMSCs) using Masson’s trichrome original staining (Figures 6D, H, L) and then they were statistically analyzed (Figure 5).

Analysing the intensity of fibrotic area, we detected no significant differences in separate uterine horn layers within the different female mice groups: the total wall of uterine horn (Kruskal-Wallis; H = 1.62; df = 2; *p* = 0.45), the myometrium-endometrium (Kruskal-Wallis; H = 1.06; df = 2; *p* = 0,59), the myometrium (Kruskal-Wallis; H = 1.66; df = 2; *p* = 0.44) and the endometrium (Kruskal-Wallis; H = 0.35; df = 2; *p* = 0.84) (Figure 5). However, the highest level of fibrosis was observed in the CheI mice group (Figure 6H) in all layers of uterine horns: the total wall of uterine horn 16.9% (±8.4%), the myometrium-endometrium 14.8% (±11.3%), the myometrium 21.4% (±9.8%) and endometrium 8.4% (±8.3%) (Figure 5).

### Evaluation of the thickness of uterine horn layers and other histological changes in different female mice groups

**FIGURE 7:**
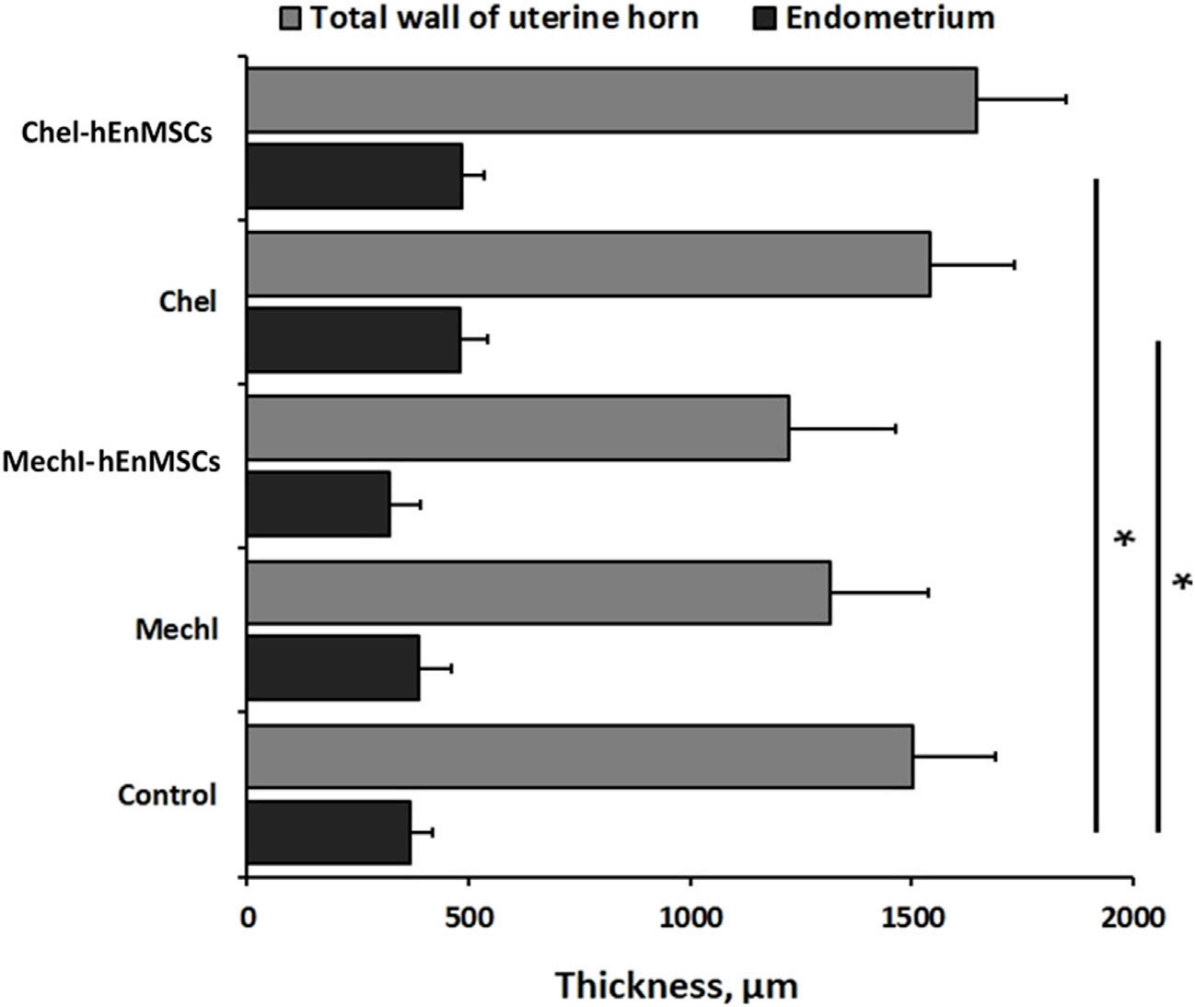
Analysis of the total wall and the endometrium thickness of uterine horns in different female mice groups: the Control mice group *(n = 4)*, the MechI mice group *(n = 6)*, the MechI-hEnMSCs mice group *(n = 4)*, the CheI mice group *(n = 3)* and the CheI-hEnMSCs mice group (n = 3). Results are presented as mean and ±S.D. Statistical significance was evaluated using the Kruskal-Wallis test and Bonferroni test, where *denotes *p* ≤ 0.05.

An evaluation of the thickness of different uterine horns layers (the total wall of uterine horn and the endometrium) was performed in the Control mice group (Figure 4B; Figure 6B), mechanically injured endometrium model (Figures 4F, 4J) and chemotherapy-induced model (Figures 6F, J) using Masson’s trichrome original staining and, after that, statistically analyzed (Figure 7).

During the study, it was revealed that the widest total wall thickness of uterine horns was observed in the Che-EnMSCs mice group (1645.3 ± 203.0 µm) (Figure 6J), a slightly lower thickness was detected in the CheI group (1539.7 ± 190.5 µm) (Figure 6F) and the Control mice group (1500.7 ± 189.7 µm) (Figure 6B; Figure 7). Overall, the total wall thickness was significantly smaller in the MechI mice group (1315.1 ± 222.0 µm) (Figure 4F) and the MechI-EnMSCs mice group (1220.8 ± 241.5 µm) (Figure 4J; Figure 7).

An evaluation of the endometrial area in all female mice groups revealed that the widest endometrial area, as well as the total wall thickness of uterine horns, was observed in the Che-EnMSCs mice group (485.1 ± 50.0 µm) (Figure 6J; Figure 7). In contrast, the result of the Control mice group (367.2 ± 50.6 µm) (Figure 4B), the MechI mice group (388.0 ± 72.6 µm) (Figure 4F) and the MechI-hEnMSCs mice group (320.5 ± 69.3 µm) (Figure 4J) showed a thickening of the narrower endometrial area (Figure 7). It was also seen that the endometrial diameter in all female mice groups was significantly reduced compared to the total wall thickness (in all cases Mann-Whitney U; *p* ≤ 0.05).

The nonparametric one-way analysis of variance revealed that the endometrial thickness of the Control mice group (Figure 6B) and the CheI mice group (Figure 6J) was statistically significant (Kruskal-Wallis; H = 6.56; df = 2; *p* = 0.04) and, moreover, the endometrial thickness in the Control group (Figure 6B) was significantly narrow in comparison with the CheI mice group (Figure 6F) and the Che-EnMSCs mice group (both Bonferroni test; *p* < 0.05) (Figure 7). Contrary to the endometrial area, the total wall thickness of uterine horns was not statistically different between the Control mice group (Figure 6B), the CheI mice group (Figure 6F) and the Che-EnMSCs mice group (Kruskal-Wallis; H = 1.62; df = 2; *p* = 0.45) (Figure 6J; Figure 7). There was also a similarity between the Control mice group (Figure 4B), the MechI mice group (Figure 4F) and the MechI-hEnMSCs mice group (Figure 4J) in relation to the thickness of endometrium (Kruskal-Wallis; H = 1.67; df = 2; *p* = 0.44) and the total wall thickness of uterine horns (Kruskal-Wallis; H = 3.72; df = 2; *p* = 0.16) (Figure 7).

**FIGURE 8:**
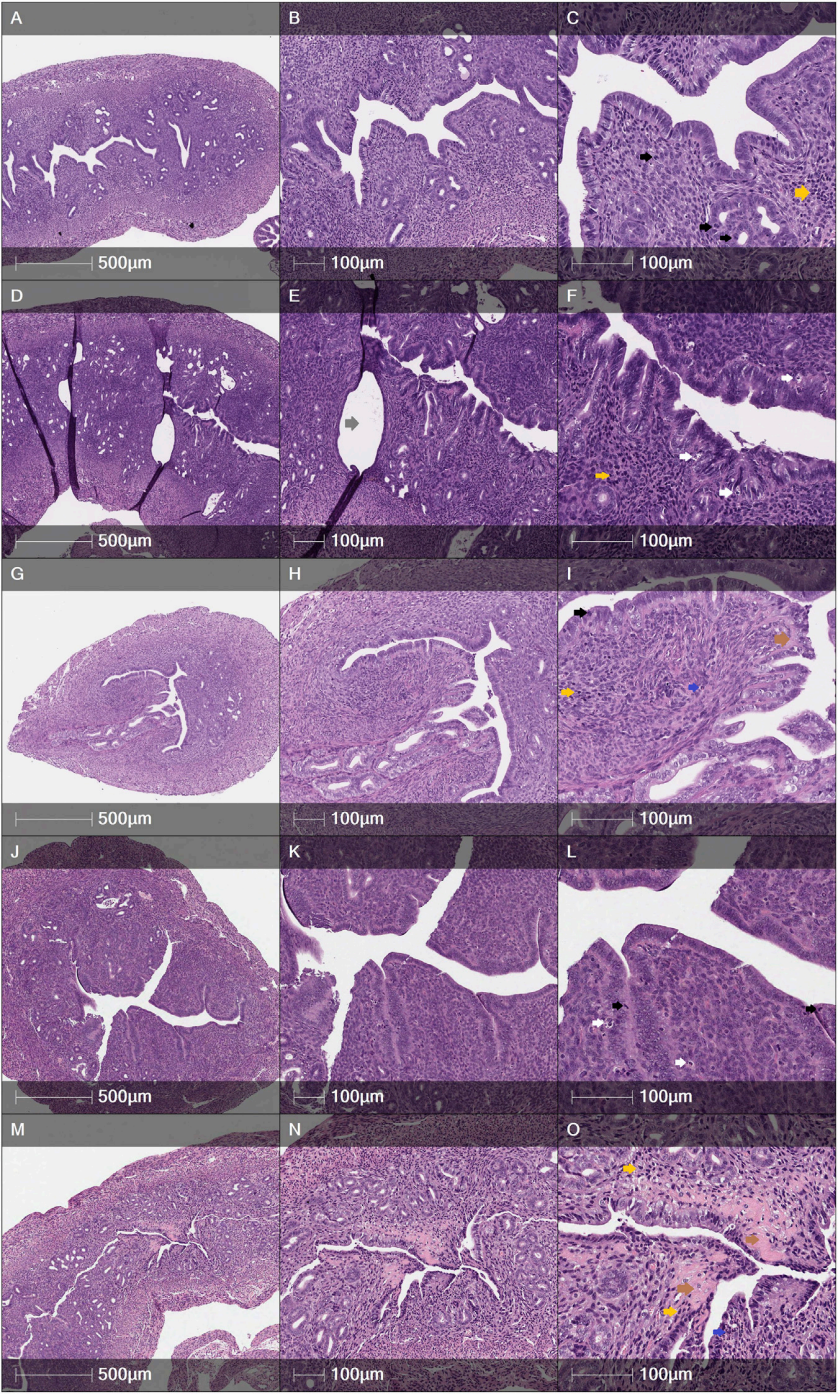
Cross sections of female mice uterine horns after H&E staining: **(A–C)**—the Control mice group, **(D–F)** - the MechI mice group, **(G–I)**—the MechI-hEnMSCs mice group, **(J–L)**—the CheI mice group, **(M–O)**—the CheI-hEnMSCs mice group. **(A–C)** Tall columnar epithelium with numerous mitotic figures in glands and stroma. Few apoptotic bodies. PMNs absent. No fibrosis. **(D–F)** Dilated glands, lined by tall columnar epithelium. Moderate apoptotic bodies, scant mitoses, moderate amount of PMNs. No fibrosis. **(G–I**) Compact endometrial stroma with few mitoses. Apoptotic bodies are scant. Few PMNs. Scant fibrosis. **(J–L)** Abundant mitosis in glands and stroma. Dense cellular endometrial stroma. Few apoptotic bodies and PMNs. No fibrosis. **(M–O)** Marked stromal fibrosis, distorted glands. Moderate mitotic figures and apoptotic bodies. Focal PMNs infiltration. Scale bar—500 μm and 100 μm. *Mitoses - black arrows; apoptotic bodies - white arrows; PMNs - blue arrows; lymphocytes - yellow arrows; fibrosis - brown arrows; dilated glands - grey arrows*.

Some tendencies could be seen according to the histology assessment by H&E, also. It was observed that in the MechI mice group, the smooth cavity of uterine horn was damaged with the detaching of the endometrium and glandular dilatation (Figures 8D–F). Meanwhile, these findings were less pronounced in the MechI-EnMSCs mice group (Figures 8G–I) and not expressed in the Control mice group (Figures 8A–C). Assessing the intensity of inflammatory cells infiltration, absent or scant number of lymphocytes and polymorphonuclear cells (PMNs) was observed in the Control mice group (Figures 8A–C) and the MechI-EnMSCs mice group (Figures 8G–I) in contrast with the MechI mice group (Figures 8D–F), where moderate amount of PMNs were seen. Evaluating results of apoptosis rate, the moderate number of apoptotic bodies in the MechI mice group (Figures 8D–F) were observed. None of these pathological changes were detected in the Control mice group (Figures 8A–C) and they showed improvement in the MechI-hEnMSCs mice group (Figures 8G–I). However, the distribution results of inflammatory cells and apoptotic bodies when comparing chemotherapy-induced mice groups (Figures 8J–O) and the Control mice group (Figures 8A–C) were quite scattered. Overall, the similar tendencies were also seen in the evaluation of mitotic bodies formation in different study’s groups (Figure 8).

### Fertility assessment in different female mice groups

A fertility assessment was performed to evaluate the direct effect of stem cell-based treatment on reproductive function in 5 different female mice groups (the Control, the MechI, the MechI-hEnMSCs, the CheI and the CheI-hEnMSCs) (Supplementary Table S1). In the mechanically injured endometrium model, we counted the number of embryo ISs only in the one uterine horn which was affected. Meanwhile, in the chemotherapy-induced female mice, the number of embryo ISs was counted in two uterine horns due to the specific effect of the medication. Chemotherapy drugs exert cytotoxic effects systemically and therefore can damage the ovaries, leading to infertility, premature ovarian failure, and, have indirect or direct deleterious effects on the uterus, that can be recognized clinically.

**FIGURE 9:**
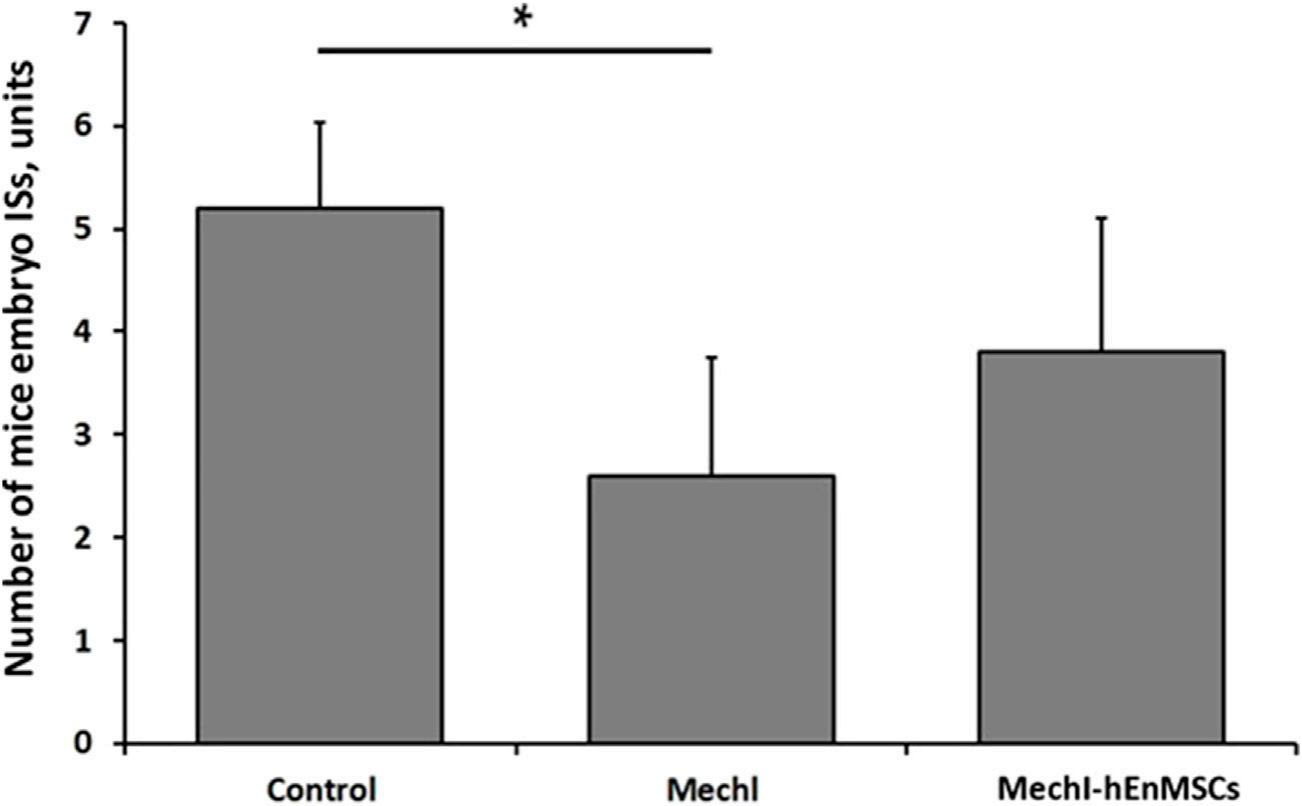
Fertility assessment in mechanically damaged endometrium model of female mice, accordingly to the number of embryo ISs in the one uterine horn: the Control mice group *(n = 5)*, the MechI mice group *(n = 5)* and the MechI-hEnMSCs mice group *(n = 5)*. Results are presented as mean and ±S.D. Statistical significance was evaluated using Kruskal-Wallis test and Bonferroni test, where *denotes *p* ≤ 0.05.

First of all, results from mechanically injured endometrium model showed a lower number of mice embryo ISs in the MechI mice group (2.6 ± 1.1 units) and the MechI-hEnMSCs mice group (3.8 ± 1,3 units) in comparison to the Control mice group (5.2 ± 0.8 units) (Figure 9). The Bonferroni *post hoc* test analysis revealed that the number of mice embryo ISs was significantly different between the Control mice group and the MechI mice group, also (Bonferroni test; *p* < 0.05) (Figure 9).

**FIGURE 10:**
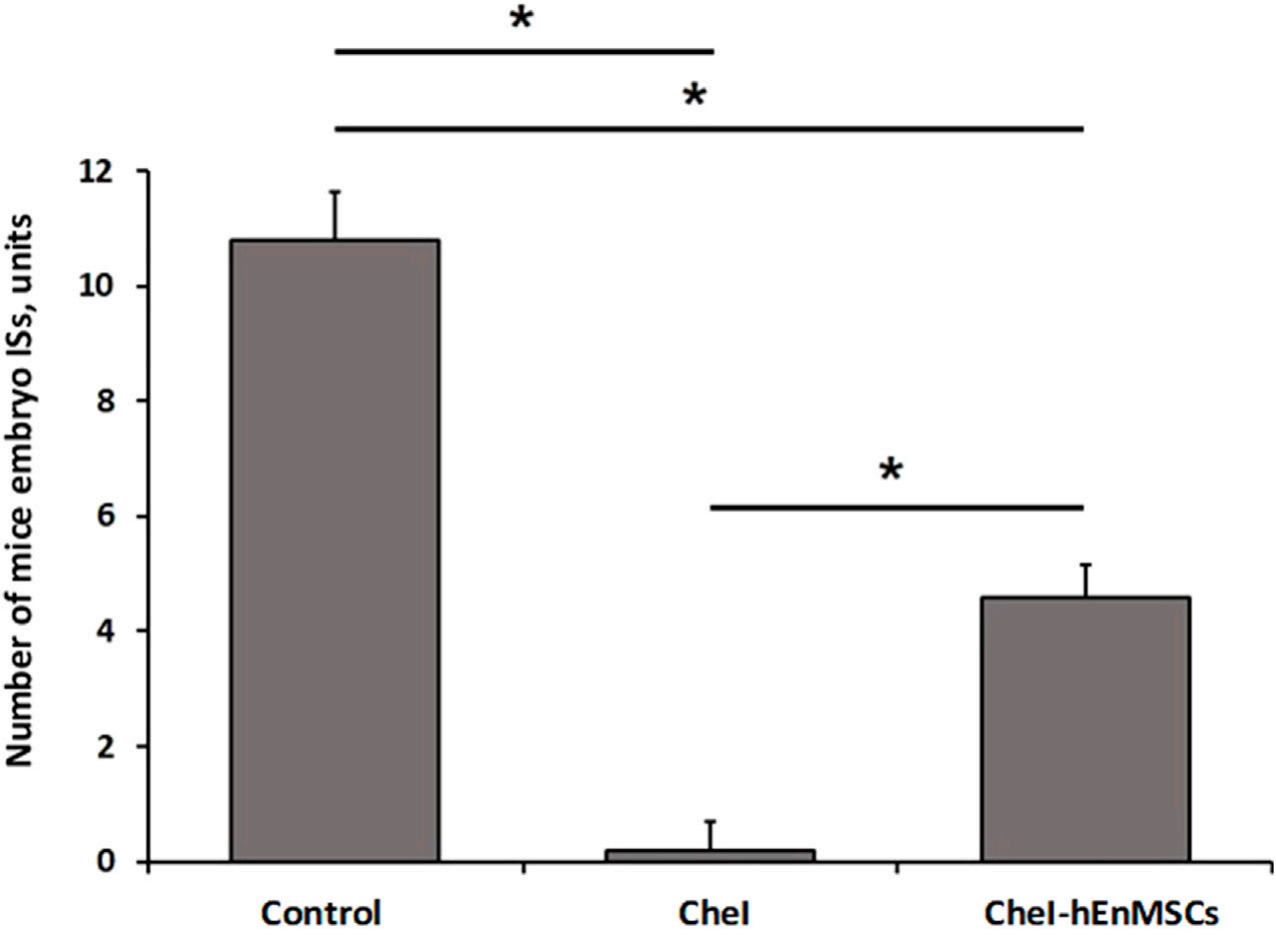
Fertility assessment in chemotherapy-induced female mice model, accordingly to the number of embryo ISs in both of uterine horns: the Control mice group *(n = 5)*, the CheI mice group *(n = 5)* and the CheI-hEnMSCs mice group *(n = 5)*. Results are presented as mean and ±S.D. Statistical significance was evaluated using Kruskal-Wallis and Bonferroni test, where *denotes *p* ≤ 0.05.

During the study of chemotherapy-induced female mice, the Control mice group was observed to have the highest number of mice embryo IS (10.8 ± 0.8 units), more than twice the number of embryo ISs was determined in the CheI-hEnMSCs mice group (4.6 ± 0.5 units) while the lowest number of embryo ISs was observed in the CheI mice group (0.2 ± 0.4 units) (Figure 10). Statistical analysis revealed that the number of embryo IS in the Control mice group, the CheI mice group and the CheI-hEnMSCs mice group was significantly different from each other (Kruskal-Wallis; H = 12.89; df = 2; *p* = 0.002; Bonferroni test; *p* < 0.001) (Figure 10).

### *Col1a1*, *Col3a1*, *Acta2* and *CD44* gene expression analysis in uterine horn tissue from different female mice groups

**FIGURE 11:**
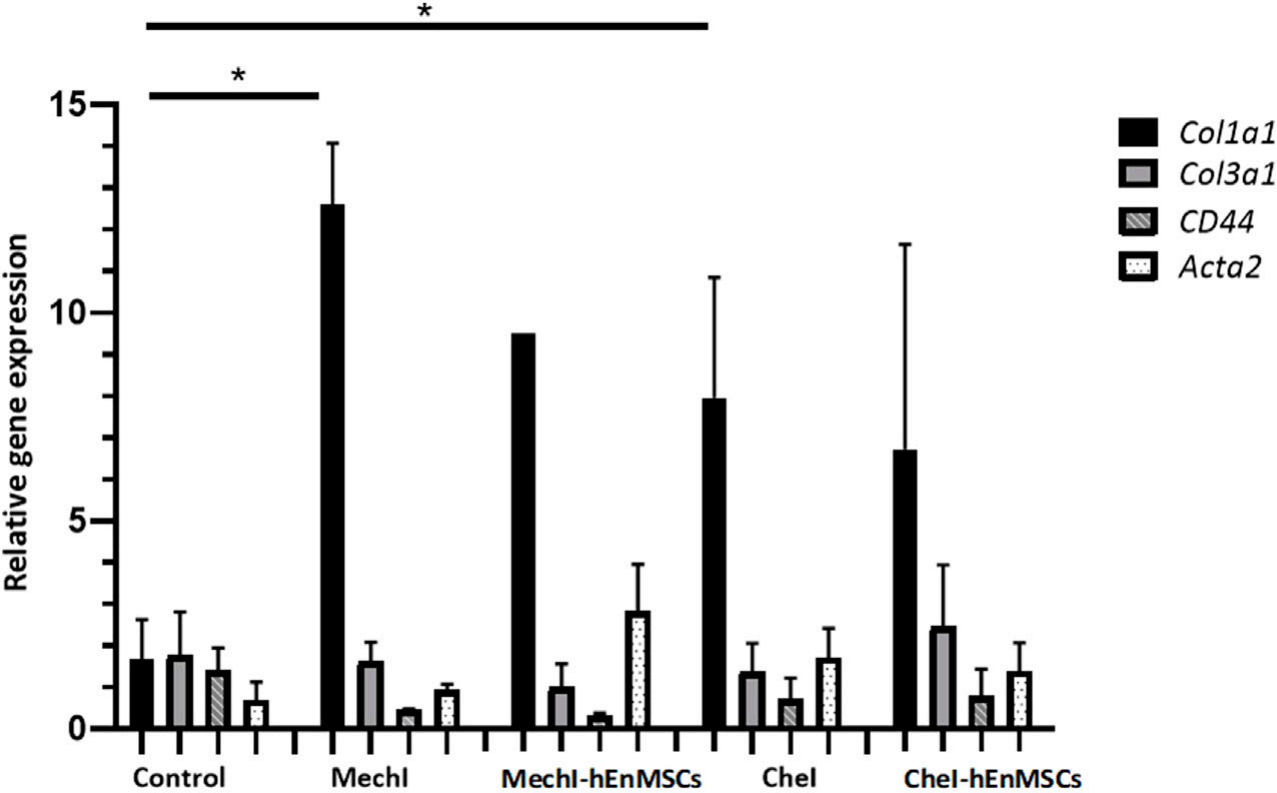
Analysis of differentially expressed *Col1a1, Col3a1, Acta2* and *CD44* genes in uterine horns samples collected from female mice groups: the Control mice group *(n = 2)*, the MechI mice group *(n = 2)*, the MechI-hEnMSCs mice group *(n = 1)*, the CheI mice group *(n = 6)* and the CheI-hEnMSCs mice group *(n = 2)* groups. Results are presented as mean and ±S.D. Relative mRNA expression was measured using RT-qPCR and results were calculated using ΔΔCt method and mRNA levels were normalized according to 18S expression. Statistical significance was evaluated using Mann–Whitney U test, where *denotes *p* ≤ 0.05.

Expression analysis of genes involved in fibrosis process was performed with 5 different female mice groups (the Control, the MechI, the MechI-hEnMSCs, the CheI and the CheI-hEnMSCs) using RNA extracted from their uterine horn tissue samples (Figure 11).

The discussed genes *Col1a1*, *Col3a1*, *Acta2* have been shown to have an association with excess fibrosis and wound healing (Liu et al., 1997; Cutroneo et al., 2007; Szóstek-Mioduchowska et al., 2019), and *CD44* gene is associated with the binding of extracellular matrix molecules, such as collagen, fibronectin and osteopontin (Foster et al., 1998). Gene expression analysis revealed that *Col1a1* gene was upregulated in all of the female mice groups receiving interventions, compared to Control mice group, although, only two of which were considered to be statistically significant (Figure 11). *Col1a1* gene was upregulated significantly in the MechI mice group and the CheI mice group (Figure 11). The expression of *Col3a1* gene throughout different group was determined to be at a very similar level to the Control mice group, although a slight upregulation can be seen in the CheI-hEnMSCs mice group when compared to the CheI group. In all groups, *CD44* was downregulated in comparison to the Control mice group while values of relative gene expression remained at a similar level between the different mice groups. *Acta2* gene was upregulated in the MechI-hEnMSCs mice group in comparison to the MechI mice group, although the results were not significant.

## Discussion

Fertility is an issue that affects many parameters of peoples’ lives, as individuals and as a couple. Nevertheless, while the main factors for the successful implantation are recognized as implantation competency of the embryo, a receptive state for the endometrium along with synchronized development for both of them, reproductive health disorders remain a significant problem (Teh et al., 2016), especially, endometrial-factor induced infertility. The lack of clear understanding of this pathology, along with conflicting theoretical and practical results from different studies have aggravated the impact of this situation to the point where couples facing this issue turn to alternatives such as surrogacy, uterine transplantation, adoption or they even choose to remain childless. Meanwhile, new strategies of diagnosis and treatment are being investigated.

Stem cell-based therapies have proved to be a promising treatment option for treating autoimmune, inflammatory, neurological, orthopaedic conditions and traumatic injuries, and could also be used for infertility. MSCs have several mechanisms of action, that could be utilized for the repair of reproductive dysfunction. The best known mode of action involves the migration of stem cells to the injured site in response to chemokines, which is followed by the differentiation into cells of the residing tissue and in the end resulting in the regeneration of the affected tissue (Wu et al., 2022). However, MSCs exhibit regenerative abilities not only through differentiation and integration, but also through paracrine signaling and immunoregulation (Jimenez-Puerta et al., 2020).

The human endometrium provides unique material in comparison to other adult tissues due to the cyclical nature of endometrial expansion, maturation and shedding, after which it again remarkably regenerates. Therefore the endometrium itself is considered to be an excellent source of stem cells and, moreover, hEnMSCs are considered to have great promise for the therapy of reproductive system disorders (Figueira et al., 2011).

The aim of this study was to deepen the investigation of the endometrial-factor induced infertility and hEnMSCs-based treatment role on it using multifactorial analysis consisted from the histological assessment, gene expression analysis and fertility assay.

First of all, in the present study we confirmed that the endometrium scratching procedure performed for female undergoing ART procedures due to couple infertility is an effective tool for the collection of stem cells. Isolated stem cells from endometrium samples, which were collected through endometrium scratching procedures, had a fibroblastic-like appearance with adherent property to the culture plate, expressed MSCs markers CD44 and CD166 and, did not express hematopoietic cell surface markers CD34, CD45 and HLA-DR. These findings fulfil the minimal criteria to define human MSCs proposed by Mesenchymal and Tissue Stem Cell Committee of the International Society for Cellular Therapy (ISCT) (Dominici et al., 2006; Machado et al., 2013; Baghaei et al., 2017). The findings negate the uncertainty about endometrium scratching usage for stem cells collection and lead us to clearly confirm that not only *stratum basalis*, but, also, *stratum functionalis* could be used for the isolation of stem cells with high proliferation, self-renewal and differentiation potential characteristics of hEnMSCs (Schwab and Gargett, 2007; Gargett and Masuda, 2010; Hu et al., 2019; Cousins et al., 2022). The evidence that hEnMSCs can be easily and repeatedly isolated without leading to major technical problems or causing inconvenience to patients—such as the need for hospitalization or anaesthesia—is worthwhile for stem cells-based therapy implementation in clinical practise (Mobarakeh et al., 2012; Stem Cell Research & Therapy | Full Text, n. d.).

Further, embryo implantation is a precise process, in which various factors come into play one after the other, so we decided to extended used models to evaluate the effectiveness of hEnMSCs therapy for induced infertility (Archibong et al., 2011; Jiang et al., 2013; Alawadhi et al., 2014; Hu et al., 2019; Stem Cell Research & Therapy | Full Text, n. d.). In this way, we combined three evaluation methods—histological assessment, gene expression analysis and fertility assay—in pursuit of a broader view which could provide new insights for understanding related mechanisms and investigate treatment strategies.

According to multivarious study results, mechanically injury of the endometrium or intraperitoneal chemotherapy was observed to be capable of causing clear negative impact on uterine tissues and, most importantly, conceiving. The fibrotic area of the total wall of uterine horns and their separate layers—the myometrium and the myometrium-endometrium was significantly wider in untreated mice group with the mechanically injured endometrium compared to the mice group with mechanically injured endometrium, which received hEnMSCs.

The same tendencies were seen in the chemotherapy-induced mice group where the highest level of fibrosis in all uterine horns’ layers was observed in the mice group which did not receive hEnMSCs in comparison with treated mice group and the Control mice group. These findings could be explained by the activity of hEnMSCs. The regeneration capacity of stromal cells is defined by the broad differentiation potential, highly efficient self-renewal, and remarkable immunomodulatory capacity and paracrine activity to migrate to the injured reproductive tissues caused by chemokines, before they differentiate and integrate somatic cells with non-tumorigenic properties (Jimenez-Puerta et al., 2020; Wiśniewska et al., 2021; Wu et al., 2022). It creates conditions for slowing down the progression of fibrosis and (or) replacing damaged tissues (Nie et al., 2011; Park et al., 2018; Wiśniewska et al., 2021). The ability of MSCs to relieve the fibrotic diseases by modulating inflammation, regenerating damaged tissues and modulating the death of stressed cells was also confirmed in the following results Qin et al., 2023.

According to the assessment of other histology elements, it was observed that hEnMSCs-based therapy can reduce the infiltration of inflammation factors such as PMNs and lymphocytes, and apoptotic bodies in uterine horns. The density of inflammatory cells and the rate of apoptotic bodies was higher in the mechanically injured female mice model, which did not receive hEnMSCs suspension, compared to the mechanically injured female mice model which received hEnMSCs suspension and the Control mice group. These repeated tendencies were also seen in fertility assessment of female mice.

During the study, the higher fertility rate was observed in harmfully affected mice groups which received stem cells therapy, even with a significant difference between the chemotherapy-induced female mice model, which did not receive stem cells and the chemotherapy-induced female mice model, which were treated.

The observed tendencies of histological changes and fertility assessment in different groups of female mice are fairly strong confirmation of the relationships between both of them and could be explained by several known physiological mechanisms.

First of all, the fibrotic endometrium is characterized by poor epithelial growth, poor vascular development, impaired endometrium function and the displacement of extracellular matrix by the fibrous connective tissues, which can lead to uterine cavity degeneration, progression to intrauterine adhesions or Asherman’s syndrome and, ultimately to embryo implantation dysfunction and consequent infertility or spontaneous abortion (Deans and Abbott, 2010; Cai et al., 2016; Bai et al., 2019). Secondly, apoptosis is a form of programmed cell death in which cells condense and fragment their nuclear material, cytoplasmic material, and then release their contents in membrane-bound apoptotic bodies (Kerr et al., 1972). In this way, apoptotic bodies may contain a wide variety of cellular components such as micronuclei, chromatin remnants, cytosol portions, degraded proteins, DNA fragments, or even intact organelles and other metabolites which could negatively affect even gene expression (Battistelli and Falcieri, 2020; Medina et al., 2020; Zeng et al., 2020). An imbalance of apoptotic bodies clearance could cause harmful exposure to the inflammatory and immunogenic contents of dying cells, ultimately causing implantation failure and consecutive pregnancy complications (Fadok et al., 1998; Savill et al., 2002; Maderna and Godson, 2003). Moreover, few studies concluded that some substance derived from PMNs may exert toxic effects on fertilized oocytes or on spermatozoa and could thus be responsible for the endometrial-factor induced infertility (Parr et al., 1967). Also, a variation of endometrial natural killer, T and B lymphocyte populations including upregulation of them, have all been proposed as contributory factors to adverse reproductive failure outcome such as repeated implantation failure and recurrent pregnancy loss (Robertson et al., 2018; Marron and Harrity, 2019; Csabai et al., 2020). However, there is still a lack of study into each subtype, including their concentrations in the endometrium and mechanisms of activity. Further research is essential not only in the inflammatory process, but also in other fields.

The measurement of the endometrial thickness and the total diameter of the uterine horns showed controversial results, especially when studying the chemotherapy-induced mice groups. The endometrial thickness in the Control mice group was significantly narrow compared with the chemotherapy-induced mice groups that nevertheless received hEnMSCs-based therapy. The total wall thickness of uterine horns was not statistically different between these three groups of female mice, although the shortest diameter was measured in the Control mice group. Moreover, the frequency of the distribution of inflammatory cells and apoptotic bodies between chemotherapy-induced mice groups and the Control mice group was quite scattered. These results are quite unexpected because it is well known that chemotherapy is one of the main cause of premature ovarian failure (Torrealday et al., 2017; Mauri et al., 2020; Spears et al., 2019) which is characterized by hypoestrogenism and, eventually, the endometrium atrophy. This limitation might be related to several factors, including the need for more time to develop premature ovarian failure. Some pre-processing issues were also agreed: during gross examination, the cross sections of uterine horns were selected as thick as possible for further paraffin-embedding procedure in order to receive well-represented cross-sections of tissues and facilitate the whole procedure. Future perspective for overcoming this limitation is applying a more standardised gross section procedure which targets for more systemic tissue sampling regardless of the thickness of uterine horns.

Meanwhile, the last step of our study was to evaluate relationships between the expression of certain genes profile and different groups of female mice. It was observed that *Col1a1* gene was upregulated in all mice groups with mechanically injured endometrium and chemotherapy-induced mice groups with the statistically significant increase in the harmfully affected mice groups without hEnMSCs therapy. *Col1a1* encodes the major component of type I collagen, the fibrillar collagen found in most connective tissues. According to the fact that gene expression is regulated by both extrinsic and intrinsic factors to the cell and interplay between them (Scitable by nature education, 2008; Kabir and O’Connor, 2019), it could be hypothesized that stem cells-based therapy can reduce or even prevent formation of excessive fibrosis through transcriptional changes. Also, interesting insights could be made after the evaluation of *Acta2* gene profile expression in different female mice groups. *Acta2* encodes smooth muscle actin - Actin alpha 2. It was seen upregulation of *Acta2* gene expression in the mechanically injured mice group which received hEnMSCs compared with the same affected mice group which was not treated. Actin proteins are essential for the cell structure, and they are also involved in the process of cell motility, integrity, and intercellular signalling (NCBI, 2023). Moreover, *Acta2* is one of 6 different actin isoforms and is involved in the contractile apparatus of smooth muscle; mutation in this gene can cause a variety of vascular diseases, such as multisystemic smooth muscle dysfunction syndrome (NCBI, 2023). In this way, downregulation of *Acta2* gene expression can be associated with the abnormal uterine contractility which may contribute to endometrium-factor induced infertility. Considering the extensive growth of the genetic and epigenetic studies, it is believed that more clarification of actual relations between stem cells activity, gene profile expression and reproductive health disorders will come.

## Conclusion

Failure to achieve a pregnancy and successful live birth due to the endometrial-factor induced infertility is one of the major challenges in the field of reproductive medicine.

Given the study results, hEnMSCs are an attractive target for endometrial-factor induced infertility. Isolated hEnMSCs have demonstrated a positive impact on the repair of mechanically injured endometrium and chemotherapy-induced endometrium with—most importantly—successful pregnancy outcomes. Moreover, it becomes apparent that multipotent MSCs can be successfully isolated from endometrium samples collected through routine gynaecological procedure such as endometrium scratching or endometrium biopsy. This finding increases the flexibility and effectiveness of MSCs collection procedure with help to decrease inconvenience to patients. Also, our study presented that a combination of multifactorial analysis could be valuable for the deeper understanding of the endometrial-factor induced infertility and, probably other endometrium pathologies.

Overall, generated results encourage the continuation of further research in order to discover new insights in this field with the hope of identifying safe and individually effective treatment strategies in mechanical injured or chemotherapy induced endometrial-factor infertility in daily clinical practise.

## Data Availability

The data and materials used and analyzed underlying this article are available from the corresponding authors on reasonable request.

## Abbreviations

Acta2, Actin alpha 2, smooth muscle; ALCAM, Activated Leukocyte Cell Adhesion Molecule; ART, Assisted reproductive technology; BSA, Bovine serum albumin; Col1a1, Collagen, type I, alpha 1; Col3a1, Collagen, type III, alpha 1; FBS, Fetal bovine serum; H&E, Hematoxylin and eosin; hEnMSCs, Human endometrium-derived mesenchymal stem/stromal cells; ICSI, Intracytoplasmic sperm injection; ISCT, The International Society for Cellular Therapy; ISs, Implantation sites; IVF, *In vitro* fertilization; LCA, Leukocyte common antigen; MHC, Major histocompatibility complex; MSCs, Mesenchymal stem/stromal cells; PBS, Phosphate-buffered saline; PMNs, Polymorphonuclear cells; RBCs, Red blood cells; RT-qPCR, Reverse transcription-quantitative polymerase chain reaction; S.D., Standard deviation.
