# Supplementary material for: Human endometrium-derived mesenchymal stem/stromal cells application in endometrial-factor induced infertility
Source: Front Cell Dev Biol. 2023 Sep 5;11:1227487. doi: 10.3389/fcell.2023.1227487 (PMC10507732; doi:10.3389/fcell.2023.1227487)
Supplement: Supplementary file 1 [file DataSheet1.docx]

***Supplementary material***


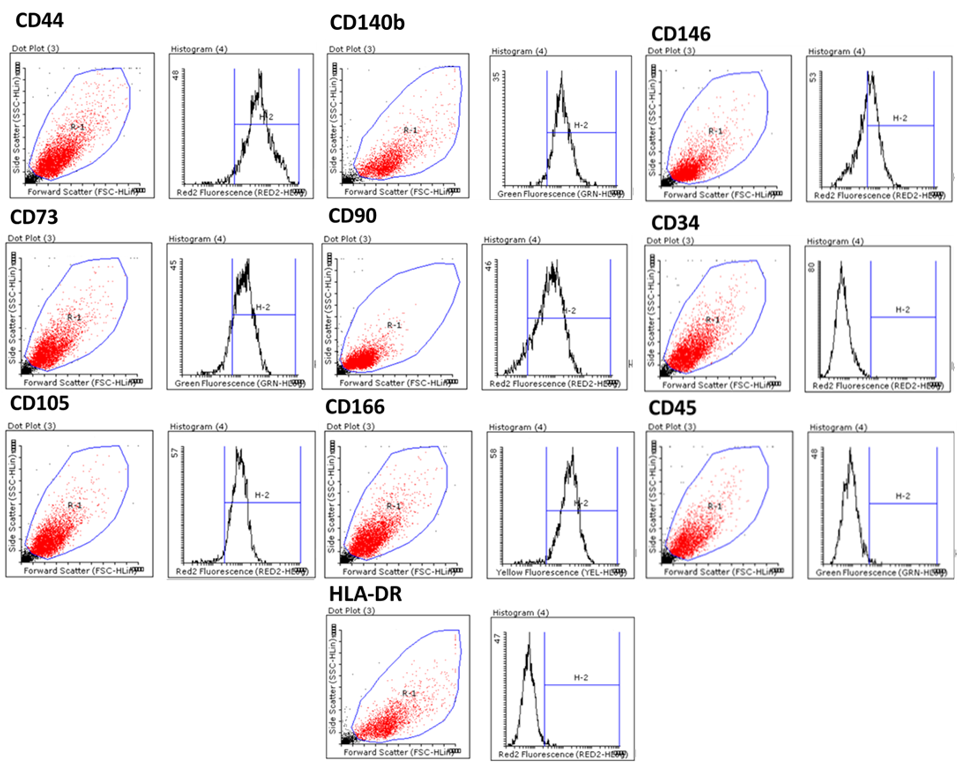
**Supplementary Figure 1.** Flow cytometry charts of different surface markers tested on hEnMSCs. The cells were tested against mesenchymal stem cells markers: CD44, CD73, CD90, CD105, CD166; endometrial stromal cells markers: CD140b, CD146; hematopoietic and immune cells markers: CD34, CD45, HLA-DR. Analysis was performed using flow cytometry.

**Supplementary Table 1.** Effect of hEnMSCs treatment on reproductive function *in vivo*. Representative images of embryo ISs from NOD.CB-17-Prkdc scid/Rj female mice in the MechI mice group, the MechI - hEnMSCs mice group, the CheI mice group, the CheI – hEnMSCs mice group and the Control mice group. These mice were sacrificed 2 weeks after the housing together with male mice. The black arrow indicated embryo ISs in the uterine horns. Embryo ISs from the MechI mice group: A left horn (undamaged) - 5, A right horn (mechanically damaged) – 4 and B left horn (undamaged) - 6, B right horn (mechanically damaged) – 3; the MechI – hEnMSCs mice group: A left horn (undamaged and not received hEnMSCs) – 4, A right horn (mechanically damaged and recieved hEnMSCs) – 5 and B left horn (undamaged and not received hEnMSCs) – 2, B right horn (mechanically damaged and recieved hEnMSCs) – 4; from the CheI mice group: A left horn – 0, B right horn – 0 and A left horn – 1, B right horn - 0; the CheI-hEnMSCs mice group: A left horn (not recieved hEnMSCs) - 1, A right horn (recieved hEnMSCs) – 3 and B left horn (not recieved hEnMSCs) - 1, A right horn (recieved hEnMSCs) – 3; from the Control mice group: A left horn - 4, A right horn – 4 and B left horn - 4, B right horn – 5.

| **MechI** | **MechI –**  **hEnMSCs** | **CheI** | **CheI – hEnMSCs** | **Control** |
| --- | --- | --- | --- | --- |
| **A** 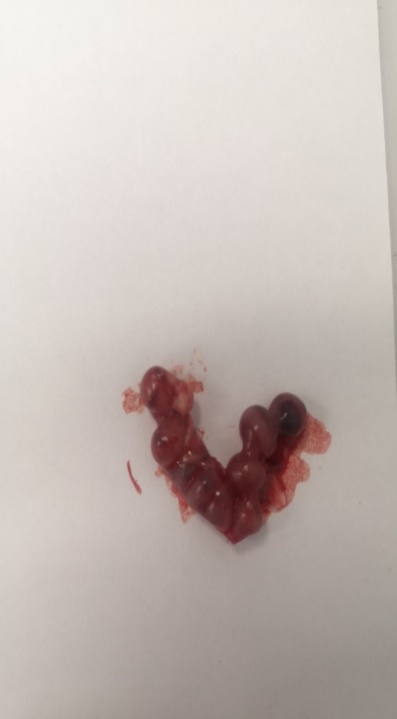 **Left horn**  **Right horn** 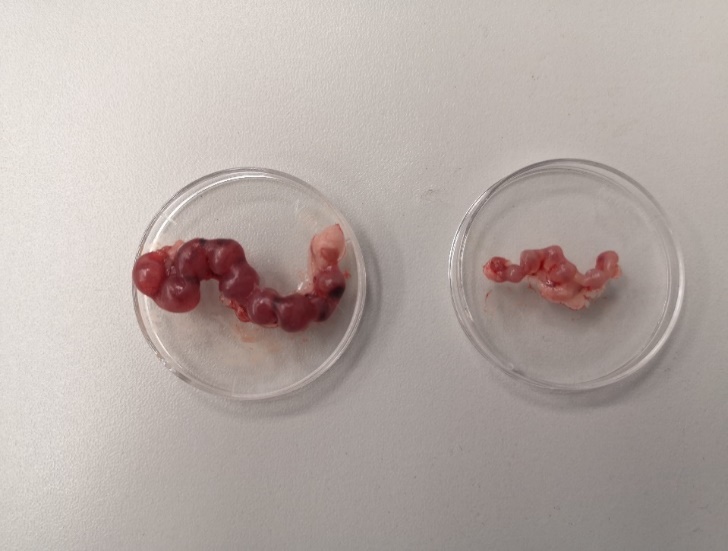 **Left horn**  **Right horn** | **A** | **A** 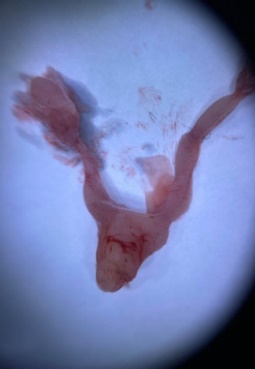 **Left horn**  **Right horn** | **A** 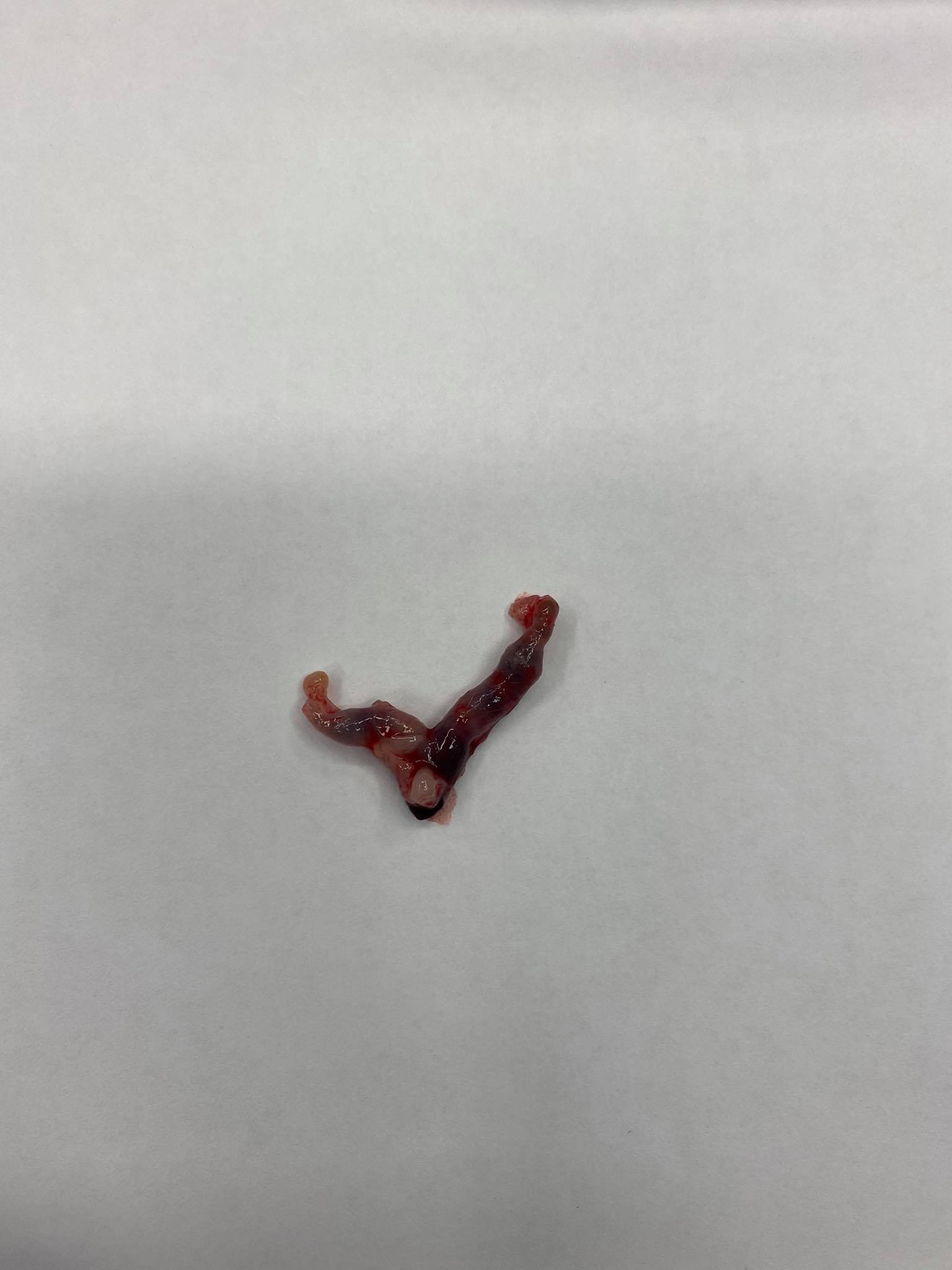 **Left horn**  **Right horn** | **A** 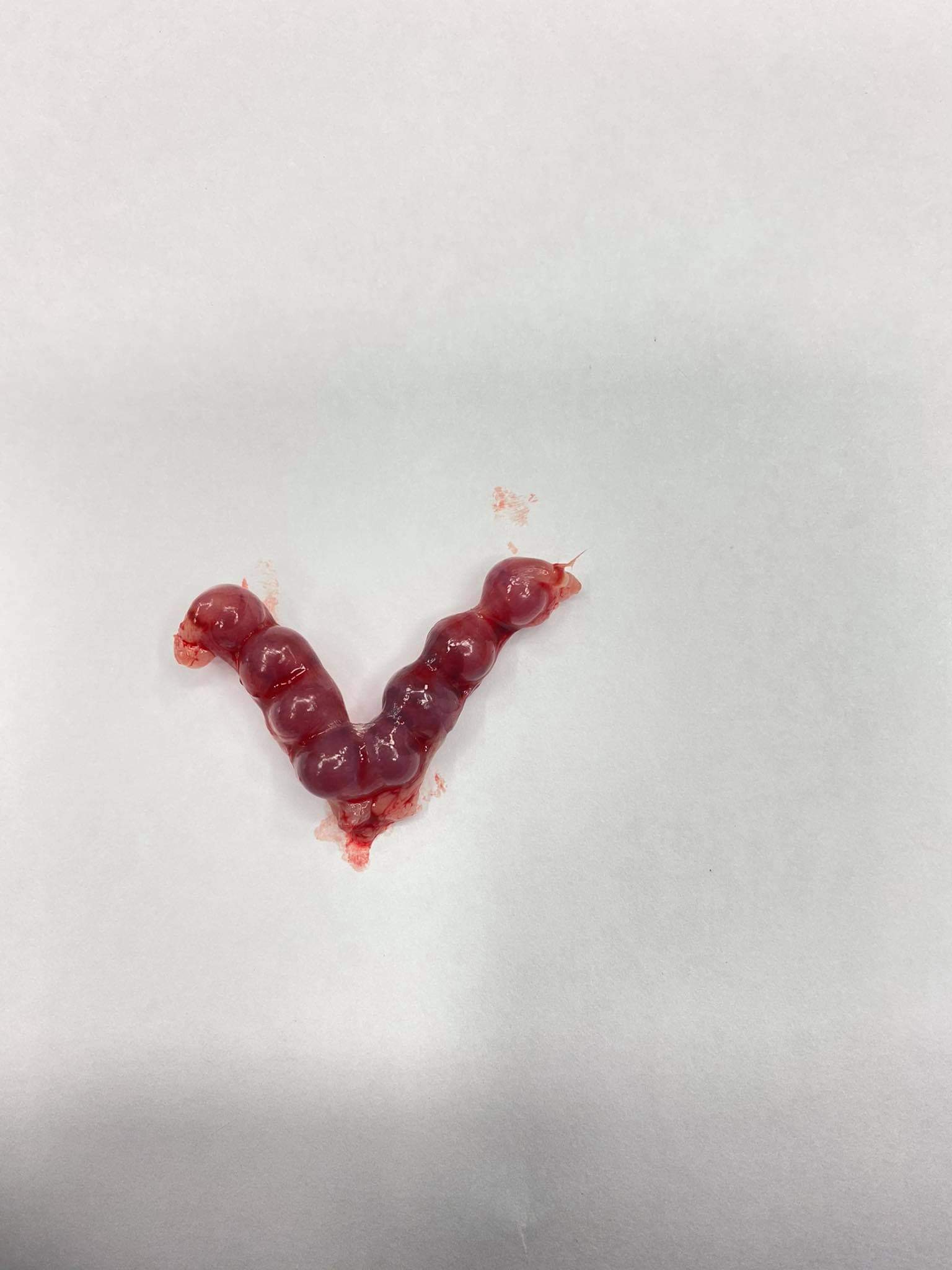 **Left horn**  **Right horn** |
| **B** 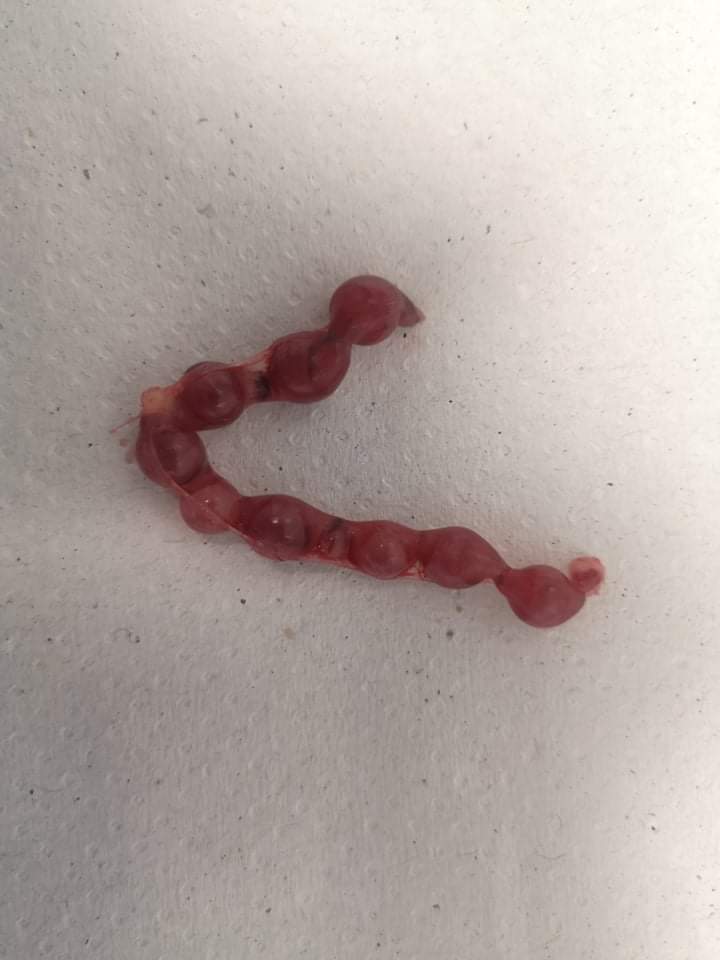 **Left horn**  **Right horn** | **B** 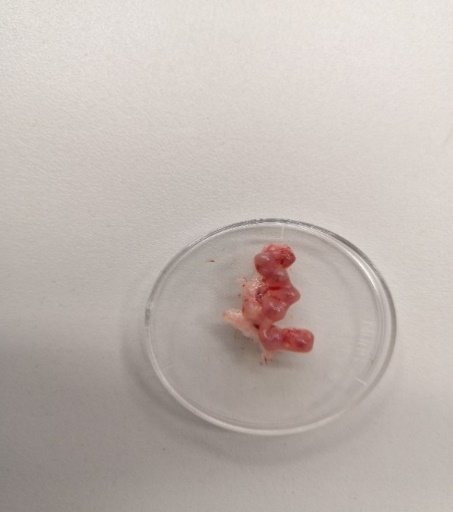 **Left horn**  **Right horn** | **B** 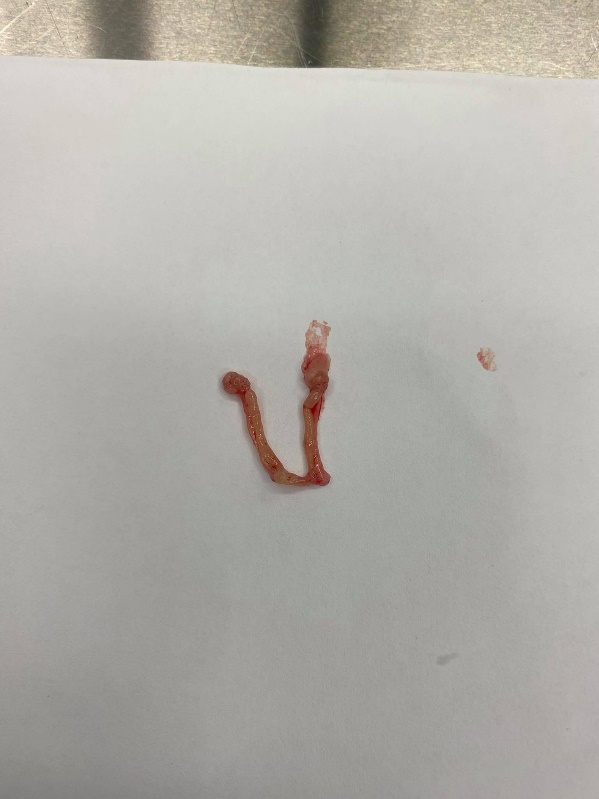 **Left horn**  **Right horn** | **B** 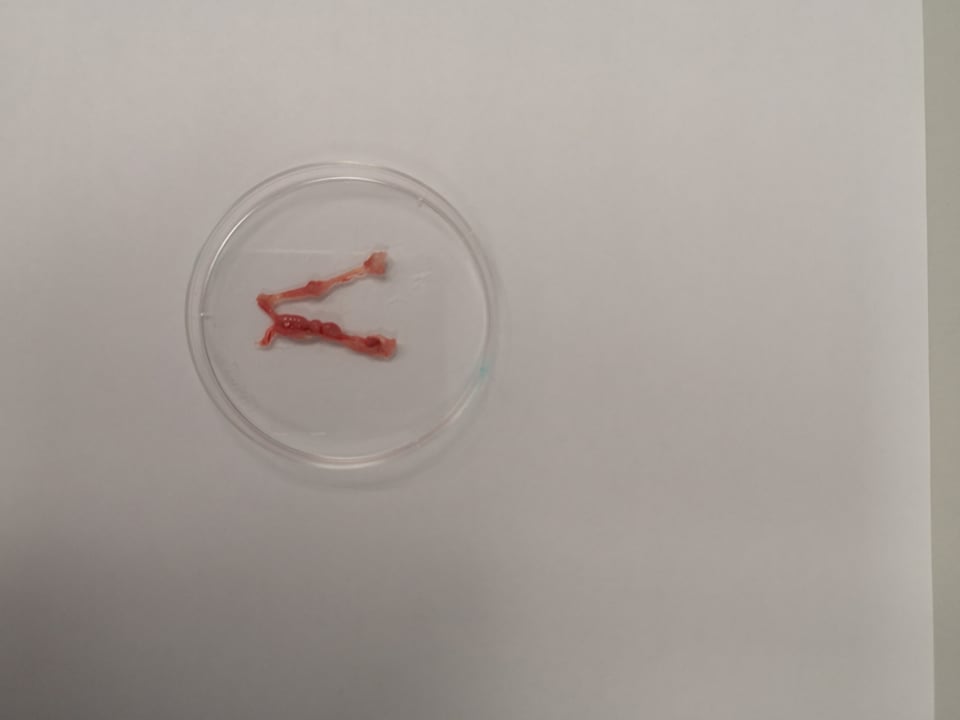 **Left horn**  **Right horn** | **B** 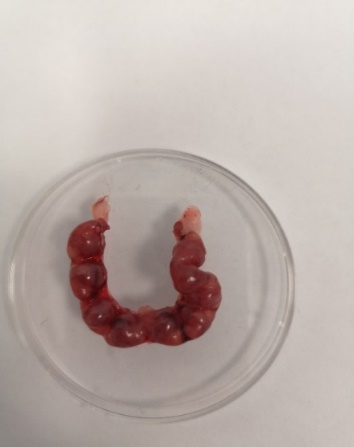 **Left horn**  **Right horn** |
